# Dynamic variability of serum sodium, potassium, and calcium and mortality after acute myocardial infarction: insights from traditional and machine learning approaches

**DOI:** 10.1186/s12872-026-05879-6

**Published:** 2026-04-24

**Authors:** Leilei Xia, Linglong Chen, Zao Jin, Zhenkun Yang, Wang Lv, Liangen Lin, Chenghao Zhang

**Affiliations:** 1 Department of Emergency, The Wenzhou Third Clinical Institute Affiliated to Wenzhou Medical University, Wenzhou People’s Hospital, Wenzhou Maternal and Child Health Care Hospital, Wenzhou People’s Hospital Affiliated to Hangzhou Medical College, No. 57, Canghou Lane, Lucheng District, Wenzhou, 325000 China; 2 Department of Breast & Thyroid Surg, Wenzhou People’s Hospital Affiliated to Hangzhou Medical College, Wenzhou People’s Hospital, The Wenzhou Third Clinical Institute Affiliated to Wenzhou Medical University, Wenzhou Maternal and Child Health Care Hospital, No. 57, Canghou Lane, Lucheng District, Wenzhou, 325000 China; 3 Institute of Clinical Medical Sciences), China-Japan Friendship Hospital, Chinese Academy of Medical Sciences & Peking Union Medical College, Beijing, 100730 China

**Keywords:** ICU mortality, Acute myocardial infarction, MIMIC-IV, Electrolyte, Coefficient of Variation

## Abstract

**Background:**

Electrolyte variability may capture dynamic homeostatic instability beyond single admission values, yet the comparative prognostic importance of sodium, potassium, and calcium variability in critically ill patients with acute myocardial infarction (AMI) remains unclear. Their relative predictive importance has not been systematically evaluated using machine learning (ML) approaches.

**Methods:**

We conducted a retrospective cohort study using MIMIC-IV (v3.1). Patients were divided into quartiles based on electrolyte coefficient of variation (CV). Associations between CV quartiles and ICU mortality (28- and 90-day) were analyzed using Cox proportional hazards models. Kaplan–Meier curves with log-rank tests compared survival across quartiles, and restricted cubic splines (RCS) examined non-linear relationships. Subgroup and interaction analyses were prespecified for age (< 65 vs. ≥65), sex, and BMI (< 30 vs. ≥30 kg/m²). Sensitivity analyses excluded early deaths (≤ 7 days), patients with CKD, and recalculated CV using the first 48 h of ICU data. Additionally, machine learning models were developed to predict 28-day mortality. The best-performing model was interpreted using Shapley Additive Explanations to identify important features.

**Results:**

In total, 3,632 ICU patients were included, median age was 70.0 years (IQR 61.0–78.0), and 65.0% were male. In Model II, higher electrolyte variability was independently associated with 28-day ICU mortality for sodium (HR = 1.11), potassium (HR = 1.02), and calcium (HR = 1.06). Using quartiles (Q1 as reference), the highest variability group (Q4) had increased risk for sodium (HR = 1.38), potassium (HR = 1.26), and calcium (HR = 2.34). For 90-day ICU mortality, Model II showed consistent associations for continuous CV: sodium (HR = 1.09), potassium (HR = 1.02), and calcium (HR = 1.06). Compared with Q1, Q4 remained significant for sodium (HR = 1.25), potassium (HR = 1.32), and calcium (HR = 1.80). RCS indicated non-linear positive relationships (*P for non-linearity* < 0.001), and calcium CV had the best discrimination. Sensitivity analyses confirmed robustness. ML analyses showed that calcium CV ranked second in importance, comparable to age, while sodium CV and potassium CV were also among the top six predictors of 28-day mortality.

**Conclusions:**

Greater sodium CV, potassium CV, and particularly calcium CV were independently associated with increased short- and medium-term ICU mortality in patients with AMI. Complementary machine learning analyses further underscore their prognostic importance for short-term ICU mortality risk stratification, supporting a clinical focus on maintaining electrolyte stability rather than solely correcting absolute levels.

**Supplementary Information:**

The online version contains supplementary material available at 10.1186/s12872-026-05879-6.

## Introduction

AMI remains a leading cause of morbidity and mortality globally, despite significant advancements in reperfusion strategies and pharmacological therapies [1]. For AMI patients admitted to the ICU, prognosis depends not only on cardiac recovery but also on the maintenance of systemic homeostasis [2]. Electrolyte disorders are among the most common complications in this population, driven by neurohormonal activation (e.g., the renin-angiotensin-aldosterone system), renal dysfunction, and the aggressive use of diuretics or vasoactive agents [2, 3]. 

Traditionally, clinical management has focused on correcting absolute electrolyte abnormalities, such as hyponatremia, hypokalemia, or hypocalcemia [4]. However, emerging evidence suggests that electrolyte variability, the fluctuation of serum levels over time, may represent a distinct and perhaps more insidious pathological marker [5]. High variability reflects a failure of homeostatic regulation and imposes significant physiological stress. Specifically, rapid fluctuations in serum sodium concentrations can induce acute osmotic stress, leading to cellular edema or shrinkage [6]. In the myocardium, this osmotic imbalance may disrupt the Na^+^/Ca^2+^ exchanger, potentially exacerbating an ischemia-reperfusion injury and promoting cellular apoptosis [7]. Fluctuations in potassium are critically linked to cardiac electrophysiology. Since the resting membrane potential of cardiomyocytes is heavily dependent on the transmembrane potassium gradient, even transient fluctuations, independent of absolute hypokalemia or hyperkalemia, can destabilize the membrane potential, reduce repolarization reserve, and lower the threshold for malignant re-entrant arrhythmias [8, 9]. Furthermore, calcium homeostasis is pivotal for myocardial excitation-contraction coupling and vascular smooth muscle tone regulation. Instability in calcium levels can directly impair myocardial contractility (inotropy) and relaxation (lusitropy), leading to hemodynamic instability [10]. Moreover, rapid shifts in calcium, particularly during reperfusion, may trigger an intracellular calcium overload (the “calcium paradox”), causing mitochondrial dysfunction and irreversible cardiomyocyte death [11]. 

Emerging evidence indicates that potassium variability is an independent predictor of in-hospital mortality, including ICU AMI cohorts and MIMIC-IV–based analyses [12]. However, studies rarely evaluate the concurrent variability of other electrolytes alongside potassium. In contrast, research on calcium in AMI has largely focused on baseline/admission serum calcium levels and their association with mortality (often non-linear or U-shaped), rather than quantifying temporal calcium variability [13, 14]. Meanwhile, in general hospitalized populations, larger changes in serum calcium during hospitalization have been associated with increased in-hospital mortality [11]. Given that ICU therapies such as regional citrate anticoagulation during continuous renal replacement therapy require frequent monitoring and protocol-driven adjustment of systemic ionized calcium, potentially producing rapid calcium shifts [15, 16], the prognostic value of calcium fluctuations in AMI warrants further investigation. It also remains unclear which electrolyte’s variability (sodium, potassium, or calcium) carries the greatest prognostic weight when modeled simultaneously.

This study utilized the large-scale Medical Information Mart for Intensive Care IV (MIMIC-IV) database to comprehensively evaluate the association between the variability of serum sodium, potassium, and calcium and 28-day and 90-day ICU mortality in patients with AMI. In addition, machine learning approaches were applied to assess the predictive value and relative importance of the variability of these electrolytes specifically for short-term (28-day) mortality risk stratification.

## Methods

### Data source

Data for this study were obtained from the MIMIC-IV (v3.1), the latest release of the database (December 19, 2024; https://mimic-iv.mit.edu/) [17]. MIMIC-IV is a publicly available critical care database developed by MIT and BIDMC and approved by the Institutional Review Boards of both institutions (BIDMC: 2001-P-001699/14; MIT: 0403000206). It contains detailed de-identified ICU patient data from admission through discharge. Leilei Xia, one of the authors, has completed Human Subjects Research Training (Certificate No. 7483634). The study adhered to the Declaration of Helsinki, and no additional ethics approval or informed consent was required because the dataset is de-identified and publicly accessible.

### Study population

This was a large retrospective cohort study in which all included cases were diagnosed as AMI using International Classification of Diseases 9 and 10 codes (specifically, ICD-9 codes: 410.x; ICD-10 codes: I21.x and I22.x). We only analyzed the first admission records for patients with multiple ICU admissions. This study used MIMIC-IV and screened 94,458 ICU admissions. We excluded patients with multiple ICU stays (*n* = 29,029), without AMI (*n* = 60,433), ICU length of stay < 1 day or > 30 days (*n* = 812), receipt of CRRT within 24 h of ICU admission (*n* = 20), and < 3 measurements of sodium/potassium/calcium (*n* = 532). The final cohort included 3,632 patients (Supplementary Figure S1).

### Data extraction

Data extraction was conducted utilizing Navicat Premium v16.3.2 to query the MIMIC-IV database. Comprehensive clinical data for all eligible AMI patients were systematically retrieved. Clinical variables encompassed the study participants’ demographic information (age, sex, race, body mass index [BMI]), Simplified Acute Physiology Score II [SAPS-II], vital signs (heart rate, systolic blood pressure [SBP], diastolic blood pressure [DBP], respiratory rate), clinical comorbidities categorized according to ICD codes (hypertension, diabetes mellitus, chronic obstructive pulmonary disease, congestive heart failure, atrial fibrillation, ventricular arrhythmia, stroke, malignant tumors, chronic kidney disease, liver disease), and interventions within the first 24 h following ICU admission (vasopressin, mechanical ventilation, diuretics, sodium supplementation, potassium supplementation, calcium supplementation). To minimize pre-analytical errors, such as sample contamination from central venous lines or ongoing intravenous infusions, electrolyte values were exclusively extracted from routine automated chemistry panels within the labevents module using specific item IDs. Measurements from point-of-care blood gas analyses were explicitly excluded. For patients with multiple measurements, the first recorded value within the initial 24 h following ICU admission was extracted.

### Exposure definition and outcomes

The mean serum sodium, potassium, and calcium levels were calculated by computing the average of all serum sodium, potassium, and calcium measurements recorded throughout the patient’s ICU stay. Total serum calcium was utilized rather than ionized calcium to preserve cohort representativeness, as ionized calcium measurements are not uniformly available for all AMI patients. To quantify the variability of sodium, potassium, and calcium, the CV was computed for each patient using all sodium, potassium, and calcium measurements obtained following ICU admission, calculated as (standard deviation/mean) × 100%. The primary outcome was the 28-day ICU mortality and 90-day ICU mortality for AMI patients.

### Statistical analysis

The proportion of missing data for each variable is reported in Supplementary Table S1. Variables with an acceptable level of missingness (< 20%) were handled using multiple imputation with the mice package in R. Briefly, several imputed datasets were generated, missing values were imputed repeatedly, and results were pooled across datasets to reduce bias and improve robustness. Normality testing indicated that all continuous variables were non-normally distributed; therefore, they are summarized as medians with interquartile ranges (IQRs), and between-group comparisons were performed using the Kruskal–Wallis test. Categorical variables are presented as counts (percentages) and were compared using Fisher’s exact test or the chi-square test, as appropriate.

In this study, participants were stratified into four groups (Q1-Q4) based on quartiles of sodium, potassium, and calcium CV. Sodium CV quartile grouping was defined as quartile 1 (Q1): Sodium CV < 1.02; quartile 2 (Q2): 1.02 ≤ Sodium CV < 1.51; quartile 3 (Q3): 1.51 ≤ Sodium CV < 2.11; quartile 4 (Q4): Sodium CV ≥ 2.11. Potassium CV quartile grouping was defined as quartile 1 (Q1): Potassium CV < 6.01; quartile 2 (Q2): 6.01 ≤ Potassium CV < 8.92; quartile 3 (Q3): 8.92 ≤ Potassium CV < 12.53; quartile 4 (Q4): Potassium CV ≥ 12.53. Calcium CV quartile grouping was defined as quartile 1 (Q1): Calcium CV < 2.28; quartile 2 (Q2): 2.28 ≤ Calcium CV < 3.64; quartile 3 (Q3):3.64 ≤ Calcium CV < 5.48; quartile 4 (Q4): Calcium CV ≥ 5.48. Two multivariable Cox proportional hazards regression models were constructed to assess the relationship between the quartiles of the CV for electrolytes and the 28/90-day ICU mortality, with the first quartile as the reference group. Hazard ratio (HR) and their 95% confidence intervals (CI) were used to express the results for the other three groups. Model I: Unadjusted; Model II: Adjusted by age, sex, race, BMI; heart rate, SBP, DBP respiratory rate, SAPS-II, hypertension, diabetes mellitus, chronic pulmonary disease, congestive heart failure, atrial fibrillation, ventricular arrhythmia, stroke, malignant tumor, chronic kidney disease, liver disease, vasopressin, mechanical ventilation, diuretics, sodium supplementation, potassium supplementation, calcium supplementation. SAPS II was included to adjust for overall baseline illness severity, while specific physiological parameters (e.g., heart rate and blood pressure) were simultaneously retained due to their direct, independent pathophysiological links to electrolyte fluctuations and cardiovascular hemodynamics in the setting of AMI. Furthermore, the cumulative incidence of 28-day and 90-day ICU mortality was analyzed using the Kaplan-Meier method, and differences in electrolyte quartiles were assessed using the Log-rank test. We performed Restricted Cubic Spline (RCS) analyses within the framework of Cox proportional hazards models to examine the relationship between the CV for sodium, potassium, and calcium and mortality outcomes. P-values for nonlinear relationships and overall significance were used to express the results.

Subgroup and interaction analyses examined the associations across key factors including admission age (< 65 vs. ≥65 years), sex, and BMI (< 30 vs. ≥30 kg/m^2^) with the mortality outcome. Interaction effects were assessed using likelihood ratio tests with *P* < 0.05 considered significant for interaction.

Several sensitivity analyses were conducted: (i) The association was reanalyzed after excluding patients who died within the first 7 days of ICU admission, in order to minimize the impact of early mortality on outcome associations; (ii) Considering that electrolytes fluctuations may be influenced by the chronic effects of pre-existing chronic kidney disease (CKD) in patients, individuals with CKD were excluded to further analyze the impact of sodium, potassium, and calcium CV on patients with renal insufficiency; (iii) To assess robustness, electrolyte variability was defined as the CV calculated using measurements obtained within the first 48 h after ICU admission; (iv) To address potential overadjustment and treatment-related bias, we performed an additional sensitivity analysis by completely removing the variables for sodium, potassium, and calcium supplementation from the multivariable Cox models.

ML analyses were performed to further evaluate the relative importance of sodium, potassium, and calcium variability in predicting 28-day ICU mortality among patients with AMI. The dataset was randomly divided into train and test sets at a ratio of 7:3. Within the train set, feature selection was performed using the Boruta algorithm to identify predictors associated with mortality.

After confirming the absence of significant correlations and multicollinearity among the selected variables, the selected variables were subsequently used to develop six ML models, including Light Gradient Boosting Machine (LightGBM), Random Forest (RF), Logistic Regression (LR), Support Vector Machine (SVM), Multilayer Perceptron (MLP), and K-Nearest Neighbors (KNN). Model hyperparameters were optimized through 5-fold cross-validation utilizing a random search approach, followed by manual fine-tuning to achieve optimal performance and prevent overfitting. Model performance was evaluated in the test set and compared with traditional SAPS II score using discrimination metrics, including receiver operating characteristic (ROC) curve analysis and the area under the curve (AUC), as well as accuracy, F1-score, and G-mean. Decision curve analysis (DCA) was further performed to evaluate the potential clinical utility of the models. Based on overall model performance, the best-performing model was selected for subsequent interpretation. Shapley Additive Explanations (SHAP) were then applied to assess variable importance and to quantify the relative contribution and ranking of sodium, potassium, and calcium variability in predicting 28-day ICU mortality.

All statistical analyses were performed using R software (v4.4.3; R Foundation for Statistical Computing, Vienna, Austria) and Python (v3.10.9; Python Software Foundation, Wilmington, DE, USA). A two-tailed *P value* < 0.05 was considered statistically significant.

## Results

### Baseline characteristics

Table 1 summarizes the baseline characteristics of the 3,632 patients included in the final analysis, stratified by 28-day ICU mortality. The cohort had a median age of 70.00 [61.00, 78.00] years and was predominantly male (65.01%). Patients with 28-day ICU mortality were significantly older than survivors (75.0 vs. 69.0 years) and had a lower BMI (26.49 vs. 27.91 kg/m²). They also exhibited higher illness severity, indicated by elevated SAPS-II scores (49.00 vs. 36.00), and a greater burden of comorbidities. Importantly, electrolyte variability was markedly higher in non-survivors across all three electrolytes (*all P* < 0.001). Similar clinical trends were observed when patients were stratified by 90-day ICU mortality (Supplementary Table S2). To contextualize the variability estimates, the median number of laboratory measurements per patient during the ICU stay was 6 (IQR: [4–9]) for sodium, 7 (IQR: [5–11]) for potassium, and 5 (IQR: [3–8]) for calcium.

**Table 1:** Baseline characteristics classified by 28-day ICU mortality

Characteristics	All (*n* = 3,632)	No 28-day ICU mortality (*n* = 2,849)	28-day ICU mortality (*n* = 783)	*P* value
Age, years	70.00 (61.00, 78.00)	69.00 (59.00, 77.00)	75.00 (65.50, 83.00)	< 0.001
Male, n (%)	2361 (65.01)	1875 (65.81)	486 (62.07)	< 0.001
Race, n (%)				0.159
White	2155 (59.33)	1714 (60.16)	441 (56.32)	
Black	277 ( 7.63)	214 (7.51)	63 (8.05)	
Hispanic	103 ( 2.84)	84 (2.95)	19 (2.43)	
Asian	84 ( 2.31)	60 (2.11)	24 (3.07)	
Others	1013 (27.89)	777 (27.27)	236 (30.14)	
Body mass index, kg/m^2^	27.66 (24.15, 32.10)	27.91 (24.43, 32.27)	26.49 (23.06, 31.28)	< 0.001
SAPS-II	38.00 (30.00, 48.00)	36.00 (28.00, 44.00)	49.00 (40.00, 58.00)	< 0.001
Vital signs				
Heart rate, bpm	85.00 (74.00, 98.00)	84.00 (74.00, 96.00)	90.00 (77.00, 103.00)	< 0.001
SBP, mmHg	118.00 (103.00, 133.00)	119.00 (104.00, 134.00)	116.00 (101.00, 132.00)	< 0.001
DBP, mmHg	66.00 (55.00, 79.00)	67.00 (56.00, 79.00)	65.00 (53.25, 77.50)	0.008
Respiratory rate, bpm	19.00 (16.00, 23.00)	18.00 (16.00, 22.00)	21.00 (18.00, 25.00)	< 0.001
Comorbidities, n (%)				
Hypertension	1189 (32.74)	997 (34.99)	192 (24.52)	< 0.001
Diabetes mellitus	1206 (33.20)	932 (32.71)	274 (34.99)	0.230
Chronic pulmonary disease	464 (12.78)	332 (11.65)	132 (16.86)	< 0.001
Congestive heart failure	1927 (53.06)	1453 (51)	474 (60.54)	< 0.001
Atrial fibrillation	1151 (31.69)	881 (30.92)	270 (34.48)	< 0.001
Ventricular arrhythmia	477 (13.13)	320 (11.23)	157 (20.05)	< 0.001
Stroke	449 (12.36)	299 (10.49)	150 (19.16)	< 0.001
Malignant tumor	339 ( 9.33)	213 (7.48)	126 (16.09)	< 0.001
Chronic kidney disease	1144 (31.50)	818 (28.71)	326 (41.63)	< 0.001
Liver disease	184 ( 5.07)	116 (4.07)	68 (8.68)	< 0.001
Intervention, n (%)				
Vasopressin	235 ( 6.47)	120 (4.21)	115 (14.69)	< 0.001
Mechanical ventilation	1129 (31.08)	758 (26.61)	371 (47.38)	< 0.001
Diuretics	1422 (39.15)	1109 (38.93)	313 (39.97)	0.594
Sodium supplementation	2682 (73.84)	2058 (72.24)	624 (79.69)	< 0.001
Potassium supplementation	894 (24.61)	731 (25.66)	163 (20.82)	0.005
calcium supplementation	689 (18.97)	526 (18.46)	163 (20.82)	0.137
Sodium CV	1.51 (1.02, 2.11)	1.46 (0.98, 2.03)	1.79 (1.25, 2.58)	< 0.001
Potassium CV	8.93 (6.01, 12.54)	8.54 (5.72, 11.89)	10.49 (7.29, 14.58)	< 0.001
Calcium CV	3.64 (2.29, 5.49)	3.37 (2.02, 5.03)	4.73 (3.09, 7.18)	< 0.001

*Abbreviations*: *CV* Coefficient of variation, *DBP* Diastolic blood pressure, *ICU* Intensive care unit, *SAPS-II* Simplified Acute Physiology Score Ⅱ, *SBP* Systolic blood pressure

Supplementary Tables S3–S5 detail patient characteristics categorized by quartiles of electrolyte variability. Across all three electrolytes, patients in the highest variability group (Q4) consistently presented with higher SAPS-II scores and increased rates of ventricular arrhythmia compared to those in Q1. As shown in Supplementary Figure S2, both ICU and in-hospital mortality rates, as well as the median lengths of stay, increased progressively across the higher quartiles for sodium, potassium, and calcium CV.

### Association between electrolyte variability and 28-day ICU mortality

Table 2 shows the association between electrolyte variability and 28-day ICU mortality. In the adjusted Model II, the continuous CV for sodium (HR = 1.11, *P* < 0.001), potassium (HR = 1.02, *P* < 0.001), and calcium (HR 1.06, *P* < 0.001) were all associated with mortality. When categorized by quartiles, compared to Q1, the Q4 group showed increased risk for sodium CV (HR = 1.38, *P* = 0.004) and potassium CV (HR = 1.26, *P* = 0.037). For calcium CV, elevated risks were observed in Q2 (HR = 1.46, *P* = 0.003), Q3 (HR 1.63, *P* < 0.001), and Q4 (HR = 2.34, *P* < 0.001).

**Table 2:** Association between electrolytes variability and 28-day ICU mortality

	Model I		Model II	
	HR (95% CI)	*P*	HR (95% CI)	*P*
Continuous Sodium CV	1.20 (1.17, 1.25)	< 0.001	1.11 (1.06, 1.17)	< 0.001
Quartiles of Sodium CV				
Q1	*reference*		*reference*	
Q2	1.25 (0.99, 1.58)	0.053	0.99 (0.78, 1.25)	0.935
Q3	1.63 (1.31, 2.03)	< 0.001	1.10 (0.87, 1.37)	0.411
Q4	2.34 (1.90, 2.87)	< 0.001	1.38 (1.11, 1.71)	0.004
Continuous Potassium CV	1.05 (1.04, 1.06)	< 0.001	1.02 (1.01, 1.04)	< 0.001
Quartiles of Potassium CV				
Q1	*reference*		*reference*	
Q2	1.08 (0.86, 1.36)	0.488	0.86 (0.68, 1.09)	0.215
Q3	1.50 (1.20, 1.86)	< 0.001	0.99 (0.79, 1.24)	0.981
Q4	2.39 (1.95, 2.92)	< 0.001	1.26 (1.02, 1.56)	0.037
Continuous Calcium CV	1.08 (1.07, 1.09)	< 0.001	1.06 (1.05, 1.08)	< 0.001
Quartiles of Calcium CV				
Q1	*reference*		*reference*	
Q2	1.65 (1.28, 2.11)	< 0.001	1.46 (1.14, 1.88)	0.003
Q3	2.09 (1.64, 2.65)	< 0.001	1.63 (1.28, 2.07)	< 0.001
Q4	3.65 (2.92, 4.56)	< 0.001	2.34 (1.85, 2.96)	< 0.001

Sodium CV: Q1 < 1.02, 1.02 ≤ Q2 < 1.51, 1.51 ≤ Q3 < 2.11, Q4 ≥ 2.11; Potassium CV: Q1 < 6.01, 6.01 ≤ Q2 < 8.92, 8.92 ≤ Q3 < 12.53, Q4 ≥ 12.53; Calcium CV: Q1 < 2.28, 2.28 ≤ Q2 < 3.64, 3.64 ≤ Q3 < 5.48, Q4 ≥ 5.48

Model I: Unadjusted

Model II: Adjusted by age, sex, race, body mass index; heart rate, systolic blood pressure, diastolic blood pressure, respiratory rate, Simplified Acute Physiology Score, hypertension, diabetes mellitus, chronic pulmonary disease, congestive heart failure, atrial fibrillation, ventricular arrhythmia, stroke, malignant tumor, chronic kidney disease, liver disease, vasopressin, mechanical ventilation, diuretics, sodium supplementation, potassium supplementation, calcium supplementation

*Abbreviations*: *CI* Confidence interval, *CV* Coefficient of variation, *HR* Hazard ratio, *ICU* Intensive care unit

**Fig. 1:**
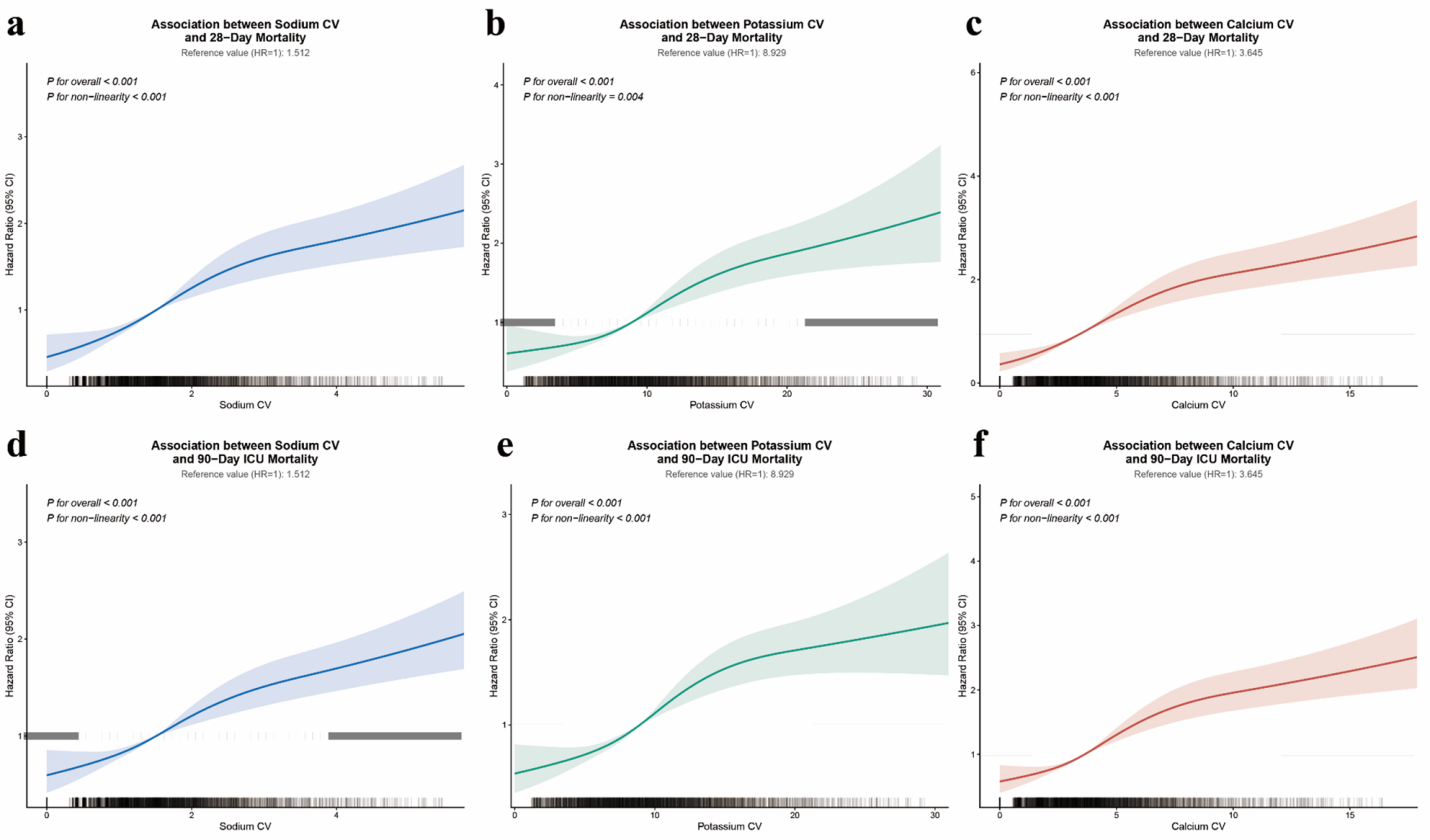
The restricted cubic spline for the relationships between sodium, potassium, and calcium CV and 28-day (**a**, **b**, **c**) and 90-day (**d**, **e**, **f**) ICU mortality. CI, confidence interval; CV, coefficient of variation; ICU, intensive care unit; HR, hazard ratio

**Fig. 2:**
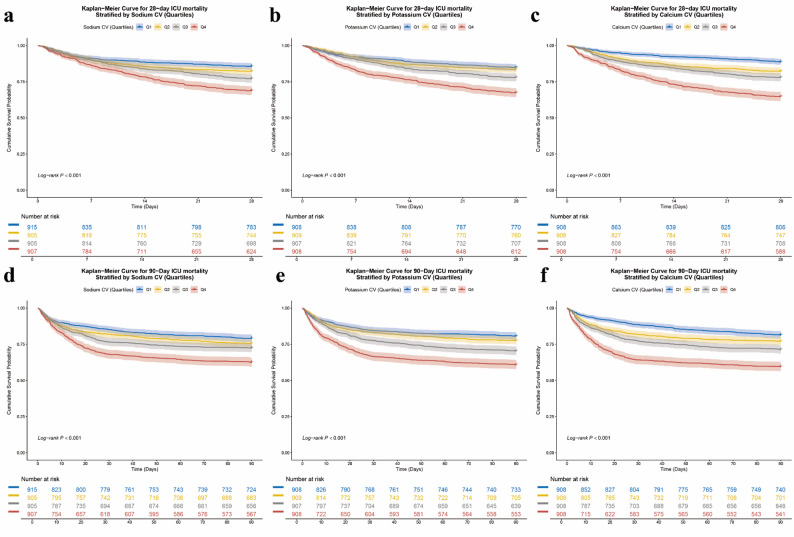
Kaplan-Meier survival curves between quartiles of sodium, potassium, and calcium CV and 28-day (**a**, **b**, **c**) and 90-day (**d**, **e**, **f**) ICU mortality. CV, coefficient of variation; ICU, intensive care unit

**Fig. 3:**
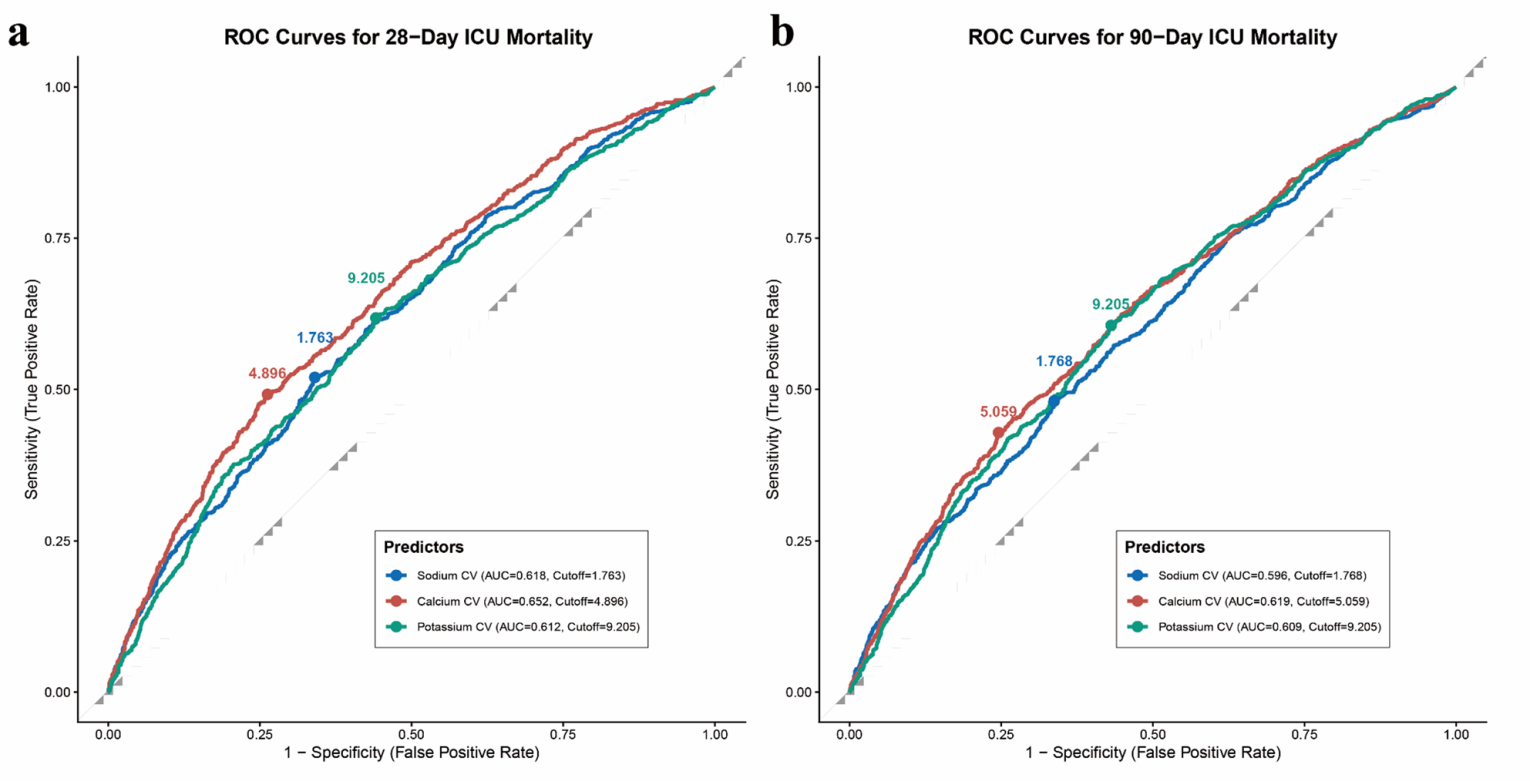
ROC curves of sodium, potassium, and calcium CV for predicting 28-day (**a**) and 90-day (**b**) ICU mortality. AUC, area under the curve; CV, coefficient of variation; ICU, intensive care unit; ROC, Receiver operating characteristic

RCS analysis confirmed a significant non-linear, positive association between the CV of all three electrolytes and 28-day ICU mortality, with hazard ratios progressively increasing as variability rose (*all P for non-linearity* < 0.001; Fig. 1a–c). Kaplan-Meier curves similarly demonstrated significantly worse cumulative survival for patients in the highest variability quartiles (Fig. 2a–c). In the predictive performance analysis, calcium CV achieved the highest AUC (0.618), followed closely by sodium and potassium CV (Fig. 3a).

### Association between electrolyte variability and 90-day ICU mortality

Table 3 presents the results for 90-day ICU mortality. In the adjusted Model II, continuous CV for sodium (HR = 1.09, *P* < 0.001), potassium (HR = 1.02, *P* < 0.001), and calcium (HR = 1.06, *P* < 0.001) were associated with mortality. Compared to the reference Q1, the Q4 group had higher hazard ratios for sodium CV (HR = 1.25, *P* = 0.018) and potassium CV (HR = 1.32, *P* = 0.005). For calcium CV, Q3 (HR = 1.37, *P* = 0.002) and Q4 (HR = 1.80, *P* < 0.001) were associated with increased mortality, while Q2 was not (HR = 1.18, *P* = 0.111). RCS and Kaplan-Meier analyses further reinforced this non-linear dose-response relationship, showing progressively declining survival rates with higher variability (Figs. 1d–f and 2d–f). For 90-day mortality prediction, calcium CV (0.619) again demonstrated the highest discriminatory ability among the electrolytes (Fig. 3b).

**Table 3:** Association between electrolytes variability and 90-day ICU mortality

	Model I		Model II	
	HR (95% CI)	*P*	HR (95% CI)	*P*
Continuous Sodium CV	1.19 (1.15, 1.23)	< 0.001	1.09 (1.04, 1.14)	< 0.001
Quartiles of Sodium CV				
Q1	*reference*		*reference*	
Q2	1.20 (0.99, 1.50)	0.062	0.98 (0.81, 1.19)	0.849
Q3	1.37 (1.14, 1.66)	< 0.001	0.96 (0.80, 1.18)	0.806
Q4	2.00 (1.68, 2.39)	< 0.001	1.25 (1.04, 1.51)	0.018
Continuous Potassium CV	1.05 (1.04, 1.06)	< 0.001	1.02 (1.01, 1.03)	< 0.001
Quartiles of Potassium CV				
Q1	*reference*		*reference*	
Q2	1.17 (0.96, 1.44)	0.112	0.96 (0.78, 1.18)	0.754
Q3	1.61 (1.33, 1.95)	< 0.001	1.14 (0.93, 1.38)	0.204
Q4	2.33(1.95, 2.79)	< 0.001	1.32 (1.08, 1.60)	0.005
Continuous Calcium CV	1.07 (1.06, 1.08)	< 0.001	1.06 (1.05, 1.07)	< 0.001
Quartiles of Calcium CV				
Q1	*reference*		*reference*	
Q2	1.29 (1.06, 1.58)	0.013	1.18 (0.96, 1.45)	0.111
Q3	1.67 (1.38, 2.04)	< 0.001	1.37 (1.13, 1.67)	0.002
Q4	2.63 (2.19, 3.16)	< 0.001	1.80 (1.48, 2.19)	< 0.001

Sodium CV: Q1 < 1.02, 1.02 ≤ Q2 < 1.51, 1.51 ≤ Q3 < 2.11, Q4 ≥ 2.11; Potassium CV: Q1 < 6.01, 6.01 ≤ Q2 < 8.92, 8.92 ≤ Q3 < 12.53, Q4 ≥ 12.53; Calcium CV: Q1 < 2.28, 2.28 ≤ Q2 < 3.64, 3.64 ≤ Q3 < 5.48, Q4 ≥ 5.48

Model I: Unadjusted

Model II: Adjusted by age, sex, race, body mass index; heart rate, systolic blood pressure, diastolic blood pressure, respiratory rate, Simplified Acute Physiology Score, hypertension, diabetes mellitus, chronic pulmonary disease, congestive heart failure, atrial fibrillation, ventricular arrhythmia, stroke, malignant tumor, chronic kidney disease, liver disease, vasopressin, mechanical ventilation, diuretics, sodium supplementation, potassium supplementation, calcium supplementation

*Abbreviations*: *CI* Confidence interval, *CV* Coefficient of variation, *HR* Hazard ratio, *ICU* Intensive care unit

### Subgroup analysis

**Fig. 4:**
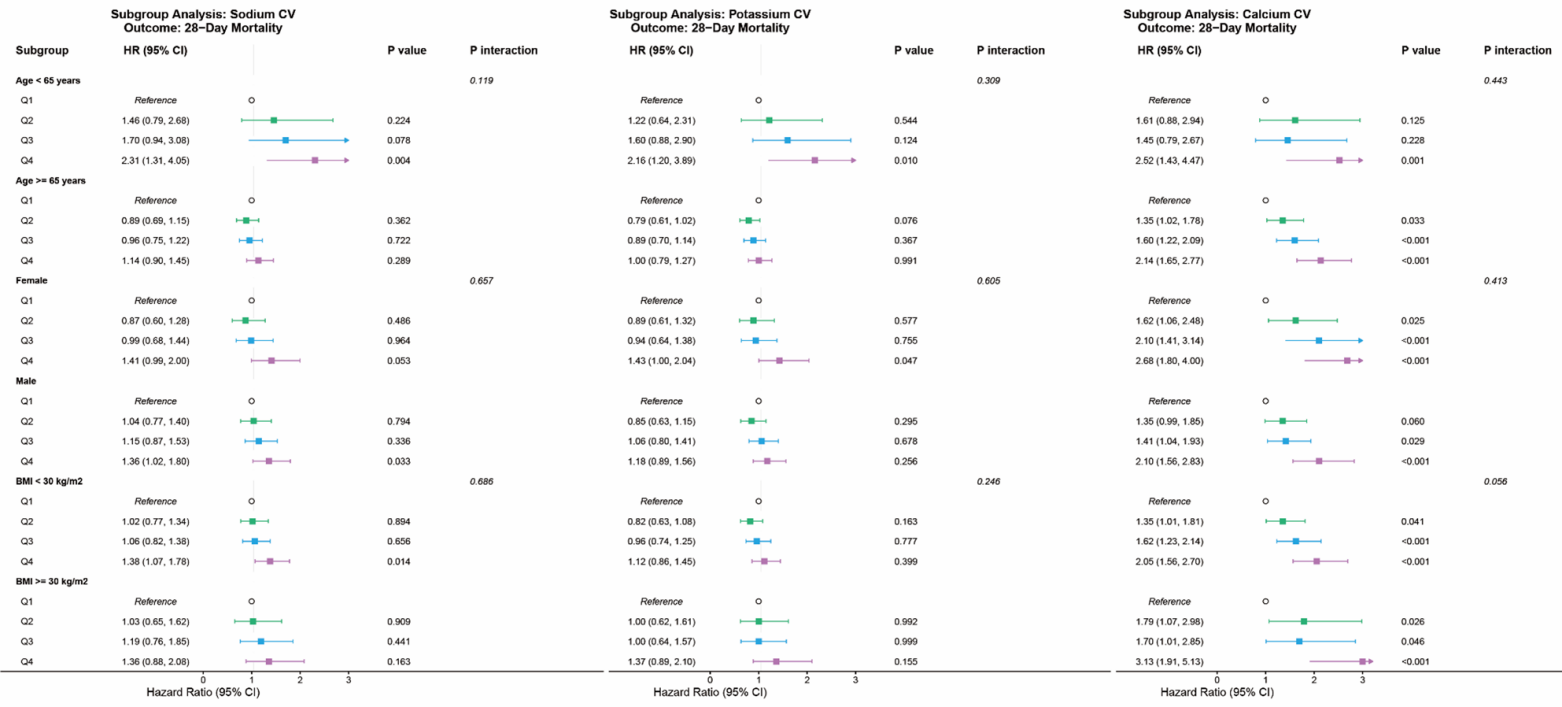
Subgroup forest plots of 28-day mortality by electrolyte CV quartiles (sodium, potassium, calcium). CI, confidence interval; CV, coefficient of variation; HR, hazard ratio

Figure 4 shows the subgroup analyses for 28-day ICU mortality. Sodium CV (Q4 vs. Q1) was associated with higher risk in patients aged < 65 years (HR = 2.31), males (HR = 1.36), and BMI < 30 kg/m² (HR = 1.38). Potassium CV (Q4 vs. Q1) was significant in patients aged < 65 years (HR = 2.16) and females (HR = 1.43). Calcium CV (Q4 vs. Q1) was consistently associated with increased risk across all subgroups (*all P* < 0.05). No significant interactions were observed (*all P for interaction* > 0.05), except a borderline interaction for calcium CV × BMI (*P* = 0.056). Subgroup trends for 90-day ICU mortality were broadly consistent, with calcium CV remaining a robust predictor across all strata (Supplementary Figure S3).

### Sensitivity analysis

Sensitivity analyses supported the robustness of the main findings. After excluding deaths within 7 days of ICU admission (Supplementary Table S6), higher sodium CV remained associated with increased 28-day and 90-day ICU mortality, both as a continuous variable (HR 1.15 and 1.11, respectively) and across quartiles, with Q4 showing higher risks than Q1 (28-day HR 2.01; 90-day HR 1.54). Similar associations were observed for potassium CV (Q4 vs. Q1: 28-day HR 1.26; 90-day HR 1.33) and calcium CV (Q4 vs. Q1: 28-day HR 2.22; 90-day HR 1.58). Excluding patients with CKD (Supplementary Table S7) or recalculating CV using values obtained within the first 48 h of ICU admission (Supplementary Table S8) yielded comparable results, with Q4 of sodium, potassium, and calcium CV remaining associated with higher 28-day and 90-day ICU mortality. To address potential overadjustment and treatment-related bias, an additional analysis was conducted excluding sodium, potassium, and calcium supplementation variables from the multivariable models (Supplementary Table S10). The associations remained statistically significant and robust, confirming the independent prognostic value of electrolyte variability regardless of direct iatrogenic interventions.

### Machine learning analysis

Feature selection using the Boruta algorithm identified 16 variables as relevant predictors of 28-day ICU mortality (Supplementary Figure S4). Sodium CV, potassium CV, and calcium CV were retained as important features and included in subsequent ML model development. Correlation and multicollinearity assessments showed no significant collinearity among the selected variables (Supplementary Figure S5).

**Fig. 5:**
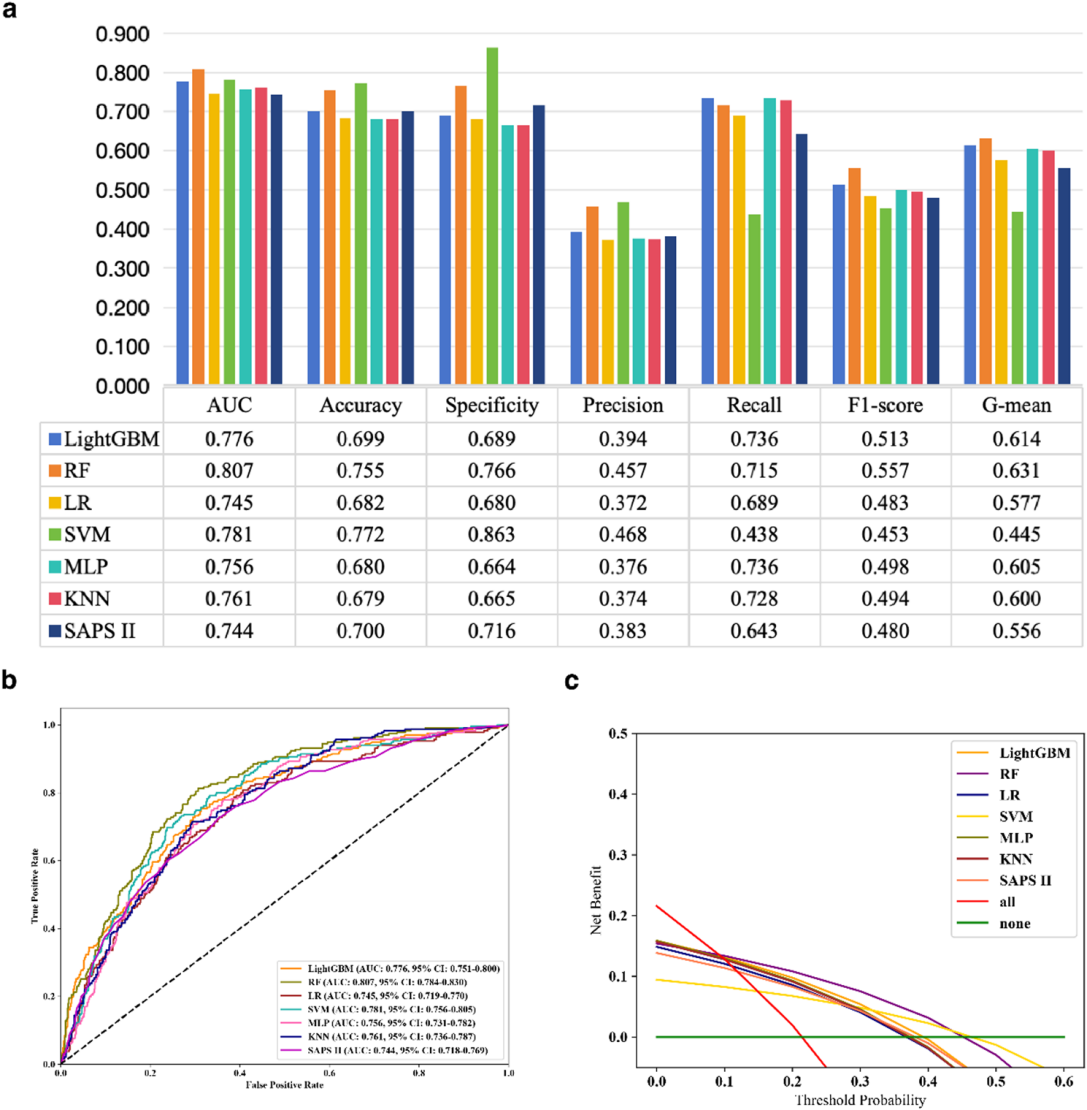
Comparison of predictive performance of machine learning models for 28-day ICU mortality in acute myocardial infarction. **a** performance metrics, (**b**) ROC curves, and (**c**) decision curve analysis. AUC, area under the receiver operating characteristic curve; DCA, decision curve analysis; G-mean, geometric mean; KNN, K-Nearest Neighbors; LightGBM, Light Gradient Boosting Machine; LR, Logistic Regression; MLP, Multilayer Perceptron; RF, Random Forest; ROC, receiver operating characteristic; SAPS II, Simplified Acute Physiology Score II; SVM, Support Vector Machine

The selected variables were subsequently entered into six ML models, including LightGBM, RF, LR, SVM, MLP, and KNN. The optimal hyperparameters for each model are presented in Supplementary Table S9. In the test set, RF demonstrated the best predictive performance, achieving the highest AUC (0.807), F1-score (0.557), and G-mean (0.631) among all models and outperforming the SAPS II score (Fig. 5a and b). DCA further confirmed the superior clinical net benefit of the RF model across a range of threshold probabilities from around 0.10 to 0.45 (Fig. 5c).

**Fig. 6:**
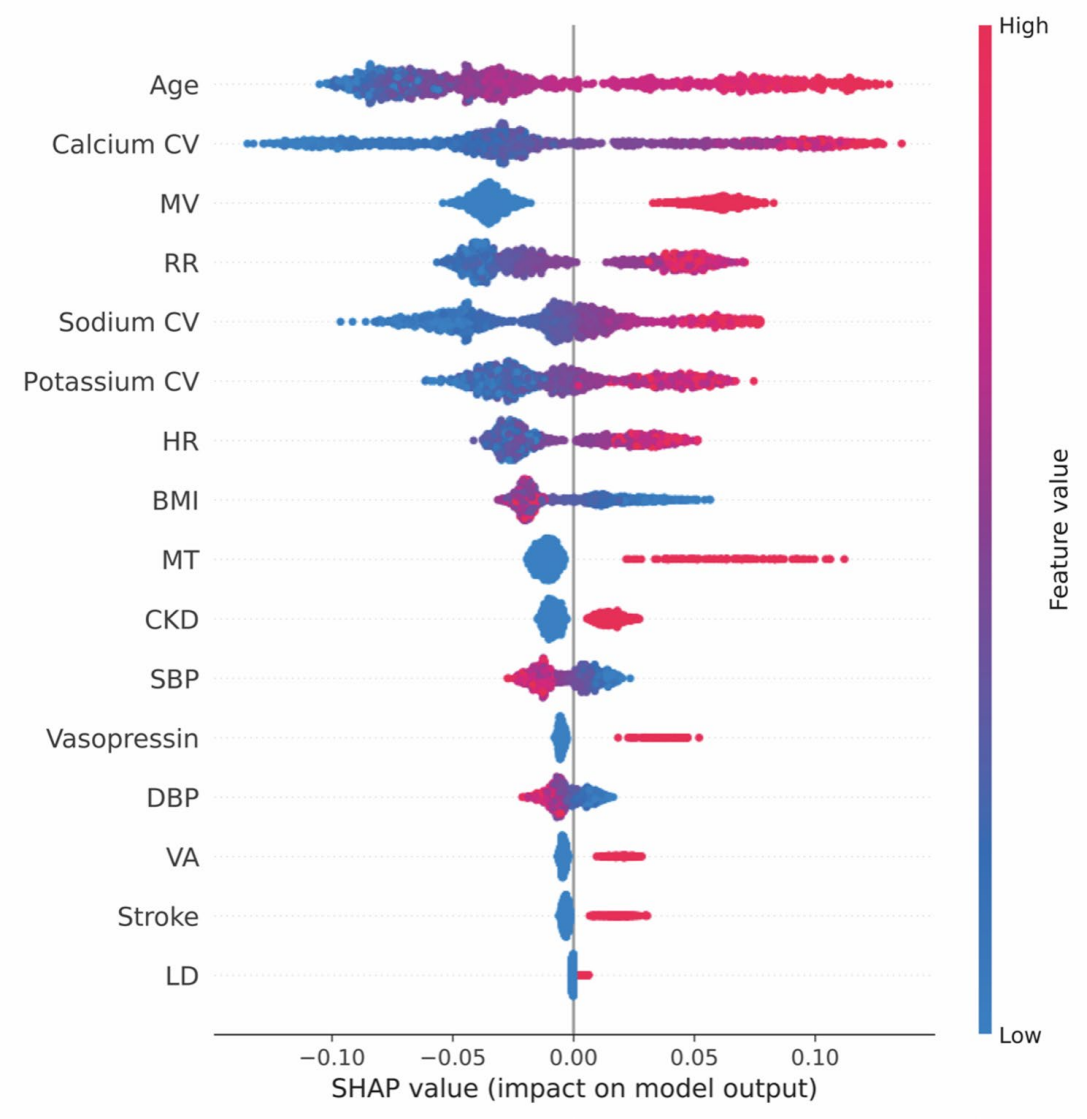
SHAP summary plot showing feature importance for predicting 28-day ICU mortality in patients with acute myocardial infarction. Each point represents an individual sample, with SHAP values indicating the impact on model output and colour representing feature values (red, high; blue, low). Features are ranked by mean absolute SHAP values. BMI, body mass index; CKD, chronic kidney disease; CV, coefficient of variation; DBP, diastolic blood pressure; HR, heart rate; LD, liver disease; MT, malignant tumor; MV, mechanical ventilation; RR, respiratory rate; SBP, systolic blood pressure; SHAP, Shapley Additive Explanations; VA, ventricular arrhythmia

SHAP analysis based on the RF model showed that calcium CV, sodium CV, and potassium CV ranked among the top six most important predictors, exceeding the contribution of most conventional clinical variables. Notably, calcium CV was the second most important predictor, with an importance level comparable to age (Fig. 6).

## Discussion

In this large retrospective cohort study of AMI patients from the MIMIC-IV database, we demonstrated that elevated variability in serum sodium, potassium, and calcium levels is significantly and independently associated with increased 28-day and 90-day ICU mortality. Notably, our results revealed a non-linear dose-response relationship between electrolyte CV and mortality risk. Among the three electrolytes, calcium variability exhibited the strongest predictive value, with patients in the Q4 facing a more than two-fold increase in 28-day mortality risk compared to those in the lowest quartile, a finding that persisted across robust sensitivity and subgroup analyses. Furthermore, complementary ML analyses consistently identified electrolyte variability as important predictors of short-term mortality. In particular, calcium CV ranked among the top predictors and showed an importance comparable to age, while sodium CV and potassium CV also demonstrated substantial contributions, exceeding those of many conventional clinical variables.

Sodium CV primarily reflects disturbances in water balance and neurohormonal regulation [18]. Rapid fluctuations in serum sodium can cause osmotic stress, leading to cellular edema or shrinkage [19], which is particularly detrimental to neurologic function and can induce systemic inflammation [20]. In the context of AMI, sodium fluctuations may also reflect the severity of heart failure and the aggressiveness of diuretic therapy. Similarly, potassium CV is a known precipitant of electrical instability [21, 22]. Since the resting membrane potential of cardiomyocytes is heavily dependent on the transmembrane potassium gradient, even transient fluctuations—independent of absolute hypokalemia or hyperkalemia—can increase myocardial vulnerability to re-entrant arrhythmias [23]. Our study confirms that high potassium CV is an independent predictor of death in AMI, likely serving as a surrogate for hemodynamic instability and renal tubular dysfunction [24]. 

Our work advances prior evidence by reframing electrolytes in AMI as dynamic homeostatic signals rather than static admission values [25]. Most AMI studies on calcium, for example, have focused on the level of calcium at presentation and reported a U-shaped association with in-hospital death. In a cohort of 11,446 AMI patients, Shiyovich et al. showed that, compared with the mid-range group (9.3–9.44 mg/dL), corrected calcium < 8.9 mg/dL and ≥ 9.86 mg/dL were associated with higher in-hospital mortality (odds ratio [OR] = 2.4 and OR = 1.7, respectively), emphasizing “too low or too high” as harmful [26]. A later AMI study using the eICU database (*N* = 7,284) refined this pattern by identifying an inflection point at 9.4 mg/dL: below 9.4 mg/dL, rising corrected calcium was protective (OR = 0.8), whereas above 9.4 mg/dL, higher calcium was associated with increased risk (OR = 1.5) [13]. Importantly, these studies still treat calcium as a single-timepoint exposure. In contrast, evidence from broader hospitalized populations suggests that change itself carries prognostic information. Thongprayoon et al. quantified calcium change as the absolute max–min difference during hospitalization and found a graded rise in mortality; after adjustment, changes of 1.0–1.4, 1.5–1.9, and ≥ 2.0 mg/dL were associated with ORs 1.55, 1.90, and 3.23, respectively, versus minimal change [27]. Building on this conceptual shift, our study operationalized electrolyte instability using the CV across ICU stay and—critically—evaluated Na, K, and Ca variability simultaneously within the same AMI-ICU cohort. While prior ICU-AMI work has already highlighted potassium variability as prognostically relevant (e.g., in an eICU AMI-ICU cohort, potassium variability ≥3rd SD carried a substantially higher in-hospital mortality risk, adjusted OR 3.3, and mean K ≥ 5.5 mmol/L was even more extreme, adjusted OR 14.8) [12], these studies generally center on potassium alone and rarely test whether other electrolyte variability contributes independently. Our data show that variability across all three electrolytes remains independently associated with mortality, but calcium variability demonstrates an earlier and steeper risk gradient: for 28-day ICU mortality, compared with Q1, calcium CV was already significant at Q2 (HR = 1.46) and rose through Q3 (HR = 1.63) to Q4 (HR = 2.34), whereas sodium and potassium variability showed more modest increases that were mainly evident in Q4 (Na: Q4 HR = 1.38; K: Q4 HR = 1.26). This “same population–same metric–same model” comparison strengthens the inference that, in the AMI-ICU setting, calcium homeostatic instability may integrate systemic severity and treatment-driven perturbations more sensitively than sodium or potassium, providing incremental prognostic information beyond traditional absolute electrolyte targets. To address potential temporal bias—wherein patients with longer ICU stays accumulate more laboratory measurements and thus potentially greater variability—we conducted a sensitivity analysis restricting the exposure window to the first 48 h of ICU admission. The prognostic significance of electrolyte variability remained robust, confirming that these fluctuations are early predictors of mortality rather than merely a byproduct of prolonged ICU stays.

Our study has several important strengths. First, it shifts the focus from “static” electrolyte levels to dynamic homeostatic instability by quantifying fluctuations with the CV across the ICU stay, thereby capturing physiologic dysregulation that may be missed by single admission measurements. This is particularly relevant because prior AMI studies on calcium largely emphasized baseline corrected/total calcium and its U-shaped relationship with in-hospital mortality [26], for example, Shiyovich et al. reported higher mortality at both low and high corrected calcium levels (vs. 9.3–9.44 mg/dL: Ca < 8.9 mg/dL OR = 2.4, Ca ≥ 9.86 mg/dL OR = 1.7) rather than temporal instability [26]. Second, unlike earlier ICU-AMI work that frequently evaluated a single electrolyte (most commonly potassium variability) our analysis directly compared sodium, potassium, and calcium variability head-to-head within the same AMI-ICU cohort [12], using the same variability metric, time window, and comprehensive adjustment set. This design allowed us to identify which electrolyte’s variability carried the greatest prognostic weight under identical modeling assumptions: calcium CV exhibited the steepest graded association with short-term mortality, with risk already increasing from Q2 and reaching a markedly elevated hazard at Q4 (28-day ICU death Q4 vs. Q1 HR = 2.34), whereas sodium and potassium CV showed more modest effects primarily at Q4 (Na: Q4 vs. Q1 HR = 1.38; K: Q4 vs. Q1 HR = 1.26). Finally, we strengthened credibility through robustness checks addressing typical critiques of variability research (early deaths, CKD, and alternative 48-hour exposure windows), and we leveraged the granular covariate structure of MIMIC-IV to adjust for severity, comorbidities, and key ICU interventions.

Importantly, most existing prognostic models for mortality prediction in critically ill AMI patients, including both conventional risk scores and machine learning–based approaches, primarily rely on baseline clinical characteristics or single time-point laboratory measurements. Dynamic changes or variability in physiological parameters are rarely incorporated into current prediction frameworks. In particular, electrolyte assessments in previous studies have largely focused on absolute levels at admission, while the potential prognostic significance of electrolyte variability has received limited attention. Our findings extend the current literature by demonstrating that fluctuations in serum sodium, potassium, and calcium provide additional prognostic information beyond baseline measurements, suggesting that dynamic monitoring of electrolyte variability may improve risk stratification and enhance existing prediction models for critically ill patients with AMI.

### Limitations

Our study has several limitations. First, due to its retrospective design, causality cannot be established. Residual confounding from unmeasured variables, such as dynamic treatment changes or patient-specific factors, may exist. Second, electrolyte variability might function as a “shadow” phenomenon reflecting disease severity or aggressive interventions rather than being a direct pathological driver. Third, CV calculations are inherently influenced by the duration and frequency of observation. Although the 48-hour sensitivity analysis mitigated this concern, surveillance bias remains possible because more severely ill patients tend to undergo more frequent laboratory testing, increasing the likelihood of capturing larger fluctuations. Finally, as a single-center study using the MIMIC-IV database, the generalizability of our findings requires validation in large-scale, multi-center prospective cohorts.

## Conclusion

High variability in serum sodium, potassium, and particularly calcium is independently associated with poor short- and medium-term survival in AMI patients. Calcium variability emerged as the most potent predictor of mortality, with a non-linear dose–response relationship observed between electrolyte variability and mortality risk. Complementary ML analyses further supported these findings, identifying electrolyte variability as a major contributor to short-term mortality prediction. Notably, calcium CV ranked as the second most important predictor, with an importance comparable to age, while potassium CV and sodium CV were also among the top six predictors, exceeding the contribution of many conventional clinical factors. These findings underscore the importance of maintaining electrolyte stability, rather than solely correcting absolute levels, in the management of critically ill AMI patients, and suggest that monitoring electrolyte variability may provide additional value for early risk stratification and clinical decision-making.

## Supplementary Information

Supplementary Material 1.

## Data Availability

In this study, the data utilized was sourced from the Medical Information Mart for Intensive Care IV (MIMIC-IV) 3.1 database. This publicly available dataset, provided by PhysioNet, comprises electronic health record data from intensive care unit patients within a large US healthcare institution. The dataset may be accessed via the following link: https://physionet.org/content/mimiciv.
