# Supplementary material for: Dynamic variability of serum sodium, potassium, and calcium and mortality after acute myocardial infarction: insights from traditional and machine learning approaches
Source: BMC Cardiovasc Disord. 2026 Apr 24;26:498. doi: 10.1186/s12872-026-05879-6 (PMC13262218; doi:10.1186/s12872-026-05879-6)
Supplement: Supplementary file 1 — Supplementary Material 1. [file 12872_2026_5879_MOESM1_ESM.docx]

Supplementary Table S1. Percentage of missing data of each variable

Supplementary Table S2. Baseline characteristics classified by 90-day ICU mortality

Supplementary Table S3. Baseline characteristics classified by quartiles of sodium CV

Supplementary Table S4. Baseline characteristics classified by quartiles of potassium CV

Supplementary Table S5. Baseline characteristics classified by quartiles of calcium CV

Supplementary Table S6. Sensitivity analysis of electrolytes variability with 28/90-day ICU mortality excluding ICU deaths within 7 days

Supplementary Table S7. Sensitivity analysis of electrolytes variability excluding chronic kidney disease patients

Supplementary Table S8. Sensitivity analysis of electrolytes variability selecting CV calculated within 48 hours of ICU admission

Supplementary Figure S1. Flowchart of the study population.

Supplementary Figure S2. ICU and hospital length of stay and mortality across electrolyte CV quartiles (Sodium, Calcium, Potassium).

Supplementary Figure S3. Subgroup forest plots of 90-day mortality by electrolyte CV quartiles (Sodium, Potassium, Calcium).

Supplementary Figure S4. Boruta feature importance ranking for predicting 28-day ICU all-cause mortality in patients with acute myocardial infarction.

Supplementary Figure S5. Pearson’s correlation test (a) and variance inflation factor test (b) for selected features.

Supplementary Table S1. Percentage of missing data of each variable

| **Characteristics** | **Missing count** | **Percentage, %** |
| --- | --- | --- |
| Body mass index, kg/m^2^ | 671/3,632 | 18.47% |
| Heart rate, bpm | 285/3,632 | 7.84% |
| SBP, mmHg | 270/3,632 | 7.43% |
| DBP, mmHg | 270/3,632 | 7.43% |
| Respiratory rate, bpm | 285/3,632 | 7.84% |

Abbreviations: SBP, systolic blood pressure; DBP, diastolic blood pressure.

Supplementary Table S2. Baseline characteristics classified by 90-day ICU mortality

| **Characteristics** | **All**  **(n = 3,632)** | **No 90-day ICU mortality**  **(n = 2,630)** | **90-day ICU mortality**  **(n = 1,002)** | ***P value*** |
| --- | --- | --- | --- | --- |
| Age, years | 70.00 (61.00, 78.00) | 68.00 (59.00, 76.00) | 75.00 (66.00, 83.00) | < 0.001 |
| Male, n (%) | 2361 (65.01) | 1743 (66.27) | 618 (61.68) | 0.009 |
| Race, n (%) |  |  |  | 0.197 |
| White | 2155 (59.33) | 1574 (59.85) | 581 (57.98) |  |
| Black | 277 ( 7.63) | 196 (7.45) | 81 (8.08) |  |
| Hispanic | 103 ( 2.84) | 83 (3.16) | 20 (2.00) |  |
| Asian | 84 ( 2.31) | 57 (2.17) | 27 (2.69) |  |
| Others | 1013 (27.89) | 720 (27.38) | 293 (29.24) |  |
| Body mass index, kg/m^2^ | 27.66 (24.15, 32.10) | 28.07 (24.60, 32.29) | 26.45 (23.04, 31.09) | < 0.001 |
| SAPS-II | 38.00 (30.00, 48.00) | 35.00 (28.00, 43.00) | 48.00 (39.00, 57.00) | < 0.001 |
| Vital signs |  |  |  |  |
| Heart rate, bpm | 85.00 (74.00, 98.00) | 83.00 (74.00, 95.00) | 89.00 (77.00, 103.00) | < 0.001 |
| SBP, mmHg | 118.00 (103.00, 133.00) | 119.25 (104.00, 134.00) | 116.00 (101.00, 132.00) | < 0.001 |
| DBP, mmHg | 66.00 (55.00, 79.00) | 67.00 (56.00, 79.00) | 65.00 (53.12, 77.00) | 0.003 |
| Respiratory rate, bpm | 19.00 (16.00, 23.00) | 18.00 (15.50, 22.00) | 21.00 (17.25, 25.00) | < 0.001 |
| Comorbidities, n (%) |  |  |  |  |
| Hypertension | 1189 (32.74) | 958 (36.43) | 231 (23.05) | < 0.001 |
| Diabetes mellitus | 1206 (33.20) | 840 (31.94) | 366 (36.53) | < 0.001 |
| Chronic pulmonary disease | 464 (12.78) | 292 (11.1) | 172 (17.17) | < 0.001 |
| Congestive heart failure | 1927 (53.06) | 1301 (49.47) | 626 (62.48) | < 0.001 |
| Atrial fibrillation | 1151 (31.69) | 798 (30.34) | 353 (35.23) | < 0.001 |
| Ventricular arrhythmia | 477 (13.13) | 287 (10.91) | 190 (18.96) | < 0.001 |
| Stroke | 449 (12.36) | 267 (10.15) | 182 (18.16) | < 0.001 |
| Malignant tumor | 339 ( 9.33) | 159 (6.05) | 180 (17.96) | < 0.001 |
| Chronic kidney disease | 1144 (31.50) | 713 (27.11) | 431 (43.01) | < 0.001 |
| Liver disease | 184 ( 5.07) | 96 (3.65) | 88 (8.78) | < 0.001 |
| Intervention, n (%) |  |  |  |  |
| Vasopressin | 235 ( 6.47) | 105 (3.99) | 130 (12.97) | < 0.001 |
| Mechanical ventilation | 1129 (31.08) | 701 (26.65) | 428 (42.71) | < 0.001 |
| Diuretics | 1422 (39.15) | 1030 (39.16) | 392 (39.12) | 0.982 |
| Sodium supplementation | 2682 (73.84) | 1902 (72.32) | 780 (77.84) | < 0.001 |
| Potassium supplementation | 894 (24.61) | 690 (26.24) | 204 (20.36) | < 0.001 |
| Calcium supplementation | 689 (18.97) | 498 (18.94) | 191 (19.06) | 0.931 |
| Sodium CV | 1.51 (1.02, 2.11) | 1.46 (0.97, 2.03) | 1.70 (1.18, 2.52) | < 0.001 |
| Potassium CV | 8.93 (6.01, 12.54) | 8.41 (5.63, 11.79) | 10.25 (7.29, 14.23) | < 0.001 |
| Calcium CV | 3.64 (2.29, 5.49) | 3.36 (2.02, 5.03) | 4.34 (2.77, 6.78) | < 0.001 |

Abbreviations: ICU, intensive care unit; SBP, systolic blood pressure; DBP, diastolic blood pressure; SAPS-II, Simplified Acute Physiology Score Ⅱ; CV, coefficient of variation.

Supplementary Table S3. Baseline characteristics classified by quartiles of sodium CV

| **Characteristics** | **Q1**  **(n = 915)** | **Q2**  **(n = 905)** | **Q3**  **(n = 905)** | **Q4**  **(n = 907)** | ***P value*** |
| --- | --- | --- | --- | --- | --- |
| Age, years | 70.00 (61.00, 79.00) | 70.00 (60.00, 78.00) | 70.00 (62.00, 78.00) | 69.00 (59.00, 77.00) | 0.099 |
| Male, n (%) | 620 (67.76) | 572 (63.2) | 601 (66.41) | 568 (62.62) | 0.061 |
| Race, n (%) |  |  |  |  | 0.219 |
| White | 573 (62.62) | 545 (60.22) | 522 (57.68) | 515 (56.78) |  |
| Black | 62 (6.78) | 72 (7.96) | 60 (6.63) | 83 (9.15) |  |
| Hispanic | 22 (2.4) | 20 (2.21) | 30 (3.31) | 31 (3.42) |  |
| Asian | 21 (2.3) | 19 (2.1) | 25 (2.76) | 19 (2.09) |  |
| Others | 237 (25.9) | 249 (27.51) | 268 (29.61) | 259 (28.56) |  |
| Body mass index, kg/m2 | 27.59 (24.34, 31.67) | 28.19 (24.24, 32.82) | 27.51 (24.17, 31.98) | 27.34 (23.72, 31.86) | 0.041 |
| SAPS-II | 35.00 (27.00, 43.00) | 38.00 (29.00, 47.00) | 40.00 (32.00, 50.00) | 42.00 (33.00, 53.00) | < 0.001 |
| Vital signs |  |  |  |  |  |
| Heart rate, bpm | 83.00 (73.00, 94.00) | 84.00 (74.00, 97.00) | 86.00 (75.00, 99.00) | 86.00 (77.00, 101.00) | < 0.001 |
| SBP, mmHg | 120.00 (105.00, 135.00) | 117.00 (103.00, 132.00) | 118.00 (102.00, 133.00) | 118.00 (103.00, 135.00) | 0.135 |
| DBP, mmHg | 67.00 (56.00, 79.00) | 65.00 (55.00, 78.00) | 66.00 (54.00, 79.00) | 67.00 (56.00, 80.00) | 0.038 |
| Respiratory rate, bpm | 18.00 (15.25, 22.00) | 19.00 (16.00, 23.00) | 19.00 (16.00, 24.00) | 20.00 (16.00, 24.00) | < 0.001 |
| Comorbidities, n (%) |  |  |  |  |  |
| Hypertension | 343 (37.49) | 297 (32.82) | 267 (29.5) | 282 (31.09) | 0.002 |
| Diabetes mellitus | 259 (28.31) | 302 (33.37) | 315 (34.81) | 330 (36.38) | 0.002 |
| Chronic pulmonary disease | 106 (11.58) | 119 (13.15) | 104 (11.49) | 135 (14.88) | 0.101 |
| Congestive heart failure | 437 (47.76) | 496 (54.81) | 502 (55.47) | 492 (54.24) | 0.003 |
| Atrial fibrillation | 281 (30.71) | 300 (33.15) | 284 (31.38) | 286 (31.53) | 0.009 |
| Ventricular arrhythmia | 96 (10.49) | 106 (11.71) | 131 (14.48) | 144 (15.88) | 0.008 |
| Stroke | 101 (11.04) | 96 (10.61) | 119 (13.15) | 133 (14.66) | 0.029 |
| Malignant tumor | 83 (9.07) | 84 (9.28) | 85 (9.39) | 87 (9.59) | 0.985 |
| Chronic kidney disease | 238 (26.01) | 298 (32.93) | 318 (35.14) | 290 (31.97) | < 0.001 |
| Liver disease | 32 (3.5) | 44 (4.86) | 41 (4.53) | 67 (7.39) | < 0.001 |
| Intervention, n (%) |  |  |  |  |  |
| Vasopressin | 23 (2.51) | 46 (5.08) | 76 (8.4) | 90 (9.92) | < 0.001 |
| Mechanical ventilation | 181 (19.78) | 261 (28.84) | 299 (33.04) | 388 (42.78) | < 0.001 |
| Diuretics | 353 (38.58) | 375 (41.44) | 356 (39.34) | 338 (37.27) | 0.324 |
| Sodium supplementation | 656 (71.69) | 639 (70.61) | 680 (75.14) | 707 (77.95) | < 0.001 |
| Potassium supplementation | 207 (22.62) | 198 (21.88) | 231 (25.52) | 258 (28.45) | 0.004 |
| Calcium supplementation | 146 (15.96) | 157 (17.35) | 189 (20.88) | 197 (21.72) | 0.004 |
| Sodium CV | 0.69 (0.49, 0.86) | 1.26 (1.12, 1.40) | 1.77 (1.62, 1.94) | 2.72 (2.37, 3.45) | < 0.001 |
| Potassium CV | 6.70 (4.46, 9.70) | 8.82 (6.05, 11.91) | 9.61 (6.47, 12.98) | 11.03 (7.91, 14.78) | < 0.001 |
| Calcium CV | 2.77 (1.62, 4.28) | 3.37 (2.18, 5.03) | 3.77 (2.45, 5.63) | 4.58 (3.17, 6.96) | < 0.001 |

Abbreviations: ICU, intensive care unit; SBP, systolic blood pressure; DBP, diastolic blood pressure; SAPS-II, Simplified Acute Physiology Score Ⅱ; CV, coefficient of variation.

Supplementary Table S4. Baseline characteristics classified by quartiles of potassium CV

| **Characteristics** | **Q1**  **(n = 908)** | **Q2**  **(n = 909)** | **Q3**  **(n = 907)** | **Q4**  **(n = 908)** | ***P value*** |
| --- | --- | --- | --- | --- | --- |
| Age, years | 71.00 (62.00, 80.00) | 69.00 (59.00, 78.00) | 70.00 (60.00, 78.00) | 70.00 (61.00, 77.00) | 0.025 |
| Male, n (%) | 619 (68.17) | 595 (65.46) | 595 (65.6) | 552 (60.79) | 0.010 |
| Race, n (%) |  |  |  |  | 0.314 |
| White | 561 (61.78) | 532 (58.53) | 551 (60.75) | 511 (56.28) |  |
| Black | 56 (6.17) | 78 (8.58) | 66 (7.28) | 77 (8.48) |  |
| Hispanic | 28 (3.08) | 24 (2.64) | 23 (2.54) | 28 (3.08) |  |
| Asian | 16 (1.76) | 22 (2.42) | 17 (1.87) | 29 (3.19) |  |
| Others | 247 (27.2) | 253 (27.83) | 250 (27.56) | 263 (28.96) |  |
| Body mass index, kg/m2 | 27.60 (24.21, 31.39) | 27.59 (24.15, 32.29) | 27.82 (24.28, 31.98) | 27.69 (23.92, 32.52) | 0.692 |
| SAPS-II | 34.00 (27.00, 42.00) | 37.00 (29.00, 45.00) | 40.00 (32.00, 49.00) | 43.00 (34.00, 54.00) | < 0.001 |
| Vital signs |  |  |  |  |  |
| Heart rate, bpm | 82.00 (72.00, 93.00) | 84.00 (74.00, 97.00) | 85.00 (75.00, 100.00) | 87.00 (77.00, 102.00) | < 0.001 |
| SBP, mmHg | 120.00 (106.00, 133.00) | 118.00 (102.00, 133.00) | 117.00 (101.50, 133.00) | 119.00 (103.00, 134.25) | 0.153 |
| DBP, mmHg | 65.00 (56.00, 78.00) | 66.00 (55.00, 78.00) | 66.00 (55.00, 80.00) | 67.00 (56.00, 80.00) | 0.547 |
| Respiratory rate, bpm | 18.00 (15.00, 22.00) | 18.00 (16.00, 22.00) | 20.00 (16.00, 24.00) | 20.00 (17.00, 24.00) | < 0.001 |
| Comorbidities, n (%) |  |  |  |  |  |
| Hypertension | 335 (36.89) | 310 (34.1) | 274 (30.21) | 270 (29.74) | 0.003 |
| Diabetes mellitus | 279 (30.73) | 302 (33.22) | 314 (34.62) | 311 (34.25) | 0.286 |
| Chronic pulmonary disease | 104 (11.45) | 101 (11.11) | 131 (14.44) | 128 (14.1) | 0.061 |
| Congestive heart failure | 443 (48.79) | 474 (52.15) | 500 (55.13) | 510 (56.17) | 0.007 |
| Atrial fibrillation | 284 (31.28) | 290 (31.9) | 307 (33.85) | 270 (29.74) | < 0.001 |
| Ventricular arrhythmia | 84 (9.25) | 99 (10.89) | 120 (13.23) | 174 (19.16) | < 0.001 |
| Stroke | 102 (11.23) | 115 (12.65) | 114 (12.57) | 118 (13) | 0.680 |
| Malignant tumor | 74 (8.15) | 79 (8.69) | 101 (11.14) | 85 (9.36) | 0.143 |
| Chronic kidney disease | 268 (29.52) | 276 (30.36) | 290 (31.97) | 310 (34.14) | 0.156 |
| Liver disease | 22 (2.42) | 47 (5.17) | 50 (5.51) | 65 (7.16) | < 0.001 |
| Intervention, n (%) |  |  |  |  |  |
| Vasopressin | 22 (2.42) | 44 (4.84) | 67 (7.39) | 102 (11.23) | < 0.001 |
| Mechanical ventilation | 158 (17.4) | 273 (30.03) | 320 (35.28) | 378 (41.63) | < 0.001 |
| Diuretics | 321 (35.35) | 368 (40.48) | 372 (41.01) | 361 (39.76) | 0.054 |
| Sodium supplementation | 650 (71.59) | 677 (74.48) | 658 (72.55) | 697 (76.76) | 0.061 |
| Potassium supplementation | 174 (19.16) | 239 (26.29) | 245 (27.01) | 236 (25.99) | < 0.001 |
| Calcium supplementation | 148 (16.3) | 161 (17.71) | 174 (19.18) | 206 (22.69) | 0.004 |
| Sodium CV | 1.11 (0.71, 1.64) | 1.45 (0.97, 2.03) | 1.60 (1.17, 2.18) | 1.86 (1.34, 2.62) | < 0.001 |
| Potassium CV | 4.23 (2.95, 5.23) | 7.48 (6.77, 8.19) | 10.53 (9.70, 11.44) | 15.71 (13.89, 19.33) | < 0.001 |
| Calcium CV | 2.68 (1.61, 4.17) | 3.30 (2.09, 5.01) | 4.00 (2.71, 5.90) | 4.52 (3.02, 7.21) | < 0.001 |

Abbreviations: ICU, intensive care unit; SBP, systolic blood pressure; DBP, diastolic blood pressure; SAPS-II, Simplified Acute Physiology Score Ⅱ; CV, coefficient of variation.

Supplementary Table S5. Baseline characteristics classified by quartiles of calcium CV

| **Characteristics** | **Q1**  **(n = 908)** | **Q2**  **(n = 908)** | **Q3**  **(n = 908)** | **Q4**  **(n = 908)** | ***P value*** |
| --- | --- | --- | --- | --- | --- |
| Age, years | 71.00 (60.00, 79.00) | 69.50 (61.00, 78.00) | 70.00 (61.00, 78.00) | 69.00 (59.00, 78.00) | 0.373 |
| Male, n (%) | 594 (65.42) | 600 (66.08) | 591 (65.09) | 576 (63.44) | 0.679 |
| Race, n (%) |  |  |  |  | 0.013 |
| White | 555 (61.12) | 566 (62.33) | 523 (57.6) | 511 (56.28) |  |
| Black | 55 (6.06) | 74 (8.15) | 81 (8.92) | 67 (7.38) |  |
| Hispanic | 27 (2.97) | 16 (1.76) | 29 (3.19) | 31 (3.41) |  |
| Asian | 25 (2.75) | 12 (1.32) | 17 (1.87) | 30 (3.3) |  |
| Others | 246 (27.09) | 240 (26.43) | 258 (28.41) | 269 (29.63) |  |
| Body mass index, kg/m2 | 27.75 (24.20, 31.77) | 28.14 (24.67, 32.38) | 27.19 (23.89, 32.03) | 27.57 (24.00, 32.14) | 0.080 |
| SAPS-II | 35.00 (27.00, 43.00) | 37.00 (29.00, 45.00) | 39.00 (31.00, 50.00) | 43.00 (33.00, 54.25) | < 0.001 |
| Vital signs |  |  |  |  |  |
| Heart rate, bpm | 83.00 (73.00, 95.00) | 84.00 (74.00, 96.00) | 85.00 (76.00, 100.00) | 87.00 (74.00, 102.00) | < 0.001 |
| SBP, mmHg | 119.00 (105.00, 134.00) | 119.00 (104.00, 134.00) | 118.00 (103.00, 133.00) | 116.00 (101.00, 133.00) | 0.036 |
| DBP, mmHg | 66.00 (56.00, 78.25) | 66.00 (56.00, 78.00) | 66.00 (55.00, 79.00) | 67.00 (54.75, 79.00) | 0.956 |
| Respiratory rate, bpm | 18.00 (15.00, 22.00) | 19.00 (16.00, 23.00) | 19.00 (16.00, 23.00) | 20.00 (16.00, 24.00) | < 0.001 |
| Comorbidities, n (%) |  |  |  |  |  |
| Hypertension | 284 (31.28) | 304 (33.48) | 322 (35.46) | 279 (30.73) | 0.120 |
| Diabetes mellitus | 298 (32.82) | 305 (33.59) | 303 (33.37) | 300 (33.04) | 0.986 |
| Chronic pulmonary disease | 109 (12) | 125 (13.77) | 106 (11.67) | 124 (13.66) | 0.406 |
| Congestive heart failure | 461 (50.77) | 489 (53.85) | 491 (54.07) | 486 (53.52) | 0.458 |
| Atrial fibrillation | 297 (32.71) | 279 (30.73) | 302 (33.26) | 273 (30.07) | 0.069 |
| Ventricular arrhythmia | 70 (7.71) | 120 (13.22) | 119 (13.11) | 168 (18.5) | 0.042 |
| Stroke | 100 (11.01) | 136 (14.98) | 111 (12.22) | 102 (11.23) | 0.039 |
| Malignant tumor | 82 (9.03) | 95 (10.46) | 86 (9.47) | 76 (8.37) | 0.478 |
| Chronic kidney disease | 294 (32.38) | 273 (30.07) | 272 (29.96) | 305 (33.59) | 0.258 |
| Liver disease | 22 (2.42) | 35 (3.85) | 46 (5.10) | 81 (8.92) | 0.055 |
| Intervention, n (%) |  |  |  |  |  |
| Vasopressin | 20 (2.2) | 45 (4.96) | 53 (5.84) | 117 (12.89) | < 0.001 |
| Mechanical ventilation | 151 (16.63) | 247 (27.2) | 324 (35.68) | 407 (44.82) | < 0.001 |
| Diuretics | 365 (40.2) | 359 (39.54) | 360 (39.65) | 338 (37.22) | 0.576 |
| Sodium supplementation | 640 (70.48) | 647 (71.26) | 673 (74.12) | 722 (79.52) | < 0.001 |
| Potassium supplementation | 183 (20.15) | 212 (23.35) | 258 (28.41) | 241 (26.54) | < 0.001 |
| Calcium supplementation | 99 (10.9) | 134 (14.76) | 205 (22.58) | 251 (27.64) | < 0.001 |
| Sodium CV | 1.18 (0.81, 1.74) | 1.43 (0.94, 1.96) | 1.62 (1.11, 2.24) | 1.82 (1.27, 2.62) | < 0.001 |
| Potassium CV | 7.00 (4.56, 10.35) | 8.27 (5.88, 11.17) | 9.41 (6.61, 12.80) | 11.14 (8.27, 14.65) | < 0.001 |
| Calcium CV | 1.52 (0.87, 1.82) | 2.94 (2.62, 3.27) | 4.37 (4.00, 4.88) | 7.42 (6.25, 9.67) | < 0.001 |

Abbreviations: ICU, intensive care unit; SBP, systolic blood pressure; DBP, diastolic blood pressure; SAPS-II, Simplified Acute Physiology Score Ⅱ; CV, coefficient of variation.

Supplementary Table S6. Sensitivity analysis of electrolytes variability with 28/90-day ICU mortality excluding ICU deaths within 7 days

|  | **28-day ICU mortality** | | **90-day ICU mortality** | |
| --- | --- | --- | --- | --- |
|  | **HR (95% CI)** | ***P*** | **HR (95% CI)** | ***P*** |
| Continuous Sodium CV | 1.15 (1.08, 1.22) | <0.001 | 1.11 (1.06, 1.18) | <0.001 |
| Quartiles of Sodium CV |  |  |  |  |
| Q1 | *reference* |  | *reference* |  |
| Q2 | 0.99 (0.71, 1.38) | 0.959 | 0.98 (0.77, 1.25) | 0.885 |
| Q3 | 1.39 (1.03, 1.89) | 0.033 | 1.06 (0.83, 1.34) | 0.654 |
| Q4 | 2.01 (1.50, 2.69) | <0.001 | 1.54 (1.23, 1.94) | <0.001 |
| Continuous Potassium CV | 1.02 (1.00, 1.04) | 0.022 | 1.02 (1.00, 1.03) | 0.022 |
| Quartiles of Potassium CV |  |  |  |  |
| Q1 | *reference* |  | *reference* |  |
| Q2 | 0.82 (0.60, 1.12) | 0.210 | 0.99 (0.78, 1.28) | 0.980 |
| Q3 | 1.12 (0.84, 1.49) | 0.445 | 1.31 (1.03, 1.65) | 0.027 |
| Q4 | 1.26 (1.02, 1.58) | 0.048 | 1.33 (1.05, 1.69) | 0.017 |
| Continuous Calcium CV | 1.07 (1.04, 1.08) | <0.001 | 1.05 (1.03, 1.07) | <0.001 |
| Quartiles of Calcium CV |  |  |  |  |
| Q1 | *reference* |  | *reference* |  |
| Q2 | 1.35 (0.98, 1.85) | 0.062 | 1.06 (0.84, 1.35) | 0.615 |
| Q3 | 1.56 (1.15, 2.14) | 0.004 | 1.30 (1.03, 1.64) | 0.028 |
| Q4 | 2.22 (1.65, 3.00) | <0.001 | 1.58 (1.26, 2.00) | <0.001 |

Sodium CV: Q1<1.02, 1.02≤Q2 < 1.51, 1.51≤Q3< 2.11, Q4≥2.11; Potassium CV: Q1<6.01,6.01≤Q2 < 8.92, 8.92≤Q3 < 12.53, Q4≥12.53; Calcium CV: Q1<2.28, 2.28≤Q2 <3.64, 3.64≤Q3 < 5.48, Q4≥5.48.

Adjusted by age, gender, race, body mass index; heart rate, systolic blood pressure, diastolic blood pressure, respiratory rate, Simplified Acute Physiology Score, hypertension, diabetes mellitus, chronic pulmonary disease, congestive heart failure, atrial fibrillation, ventricular arrhythmia, stroke, malignant tumor, chronic kidney disease, liver disease, vasopressin, mechanical ventilation, diuretics, sodium supplementation, potassium supplementation, calcium supplementation.

Abbreviations: ICU, intensive care unit; CV, coefficient of variation; HR, hazard ratio; CI, confidence interval.

Supplementary Table S7. Sensitivity analysis of electrolytes variability excluding chronic kidney disease patients

|  | **28-day ICU mortality** | | **90-day ICU mortality** | |
| --- | --- | --- | --- | --- |
|  | **HR (95% CI)** | ***P*** | **HR (95% CI)** | ***P*** |
| Continuous Sodium CV | 1.12 (1.06, 1.19) | <0.001 | 1.10 (1.04, 1.16) | <0.001 |
| Quartiles of Sodium CV |  |  |  |  |
| Q1 | *reference* |  | *reference* |  |
| Q2 | 1.00 (0.73, 1.38) | 0.974 | 0.97 (0.75, 1.28) | 0.873 |
| Q3 | 1.18 (0.86, 1.60) | 0.292 | 0.96 (0.74, 1.25) | 0.786 |
| Q4 | 1.60 (1.19, 2.13) | 0.002 | 1.36 (1.06, 1.75) | 0.014 |
| Continuous Potassium CV | 1.03 (1.02, 1.05) | <0.001 | 1.03 (1.02, 1.04) | <0.001 |
| Quartiles of Potassium CV |  |  |  |  |
| Q1 | *reference* |  | *reference* |  |
| Q2 | 0.97 (0.70, 1.35) | 0.881 | 1.09 (0.82, 1.46) | 0.532 |
| Q3 | 1.19 (0.87, 1.64) | 0.272 | 1.36 (1.03, 1.80) | 0.029 |
| Q4 | 1.60 (1.17, 2.17) | 0.003 | 1.67 (1.27, 2.20) | <0.001 |
| Continuous Calcium CV | 1.07 (1.05, 1.08) | <0.001 | 1.06 (1.04, 1.08) | <0.001 |
| Quartiles of Calcium CV |  |  |  |  |
| Q1 | *reference* |  | *reference* |  |
| Q2 | 1.66 (1.16, 2.37) | 0.005 | 1.31 (0.98, 1.76) | 0.065 |
| Q3 | 1.91 (1.35, 2.71) | <0.001 | 1.53 (1.16, 2.03) | 0.003 |
| Q4 | 2.86 (2.05, 4.01) | <0.001 | 2.16 (1.64, 2.83) | <0.001 |

Sodium CV: Q1<1.02, 1.02≤Q2 < 1.51, 1.51≤Q3< 2.11, Q4≥2.11; Potassium CV: Q1<6.01,6.01≤Q2 < 8.92, 8.92≤Q3 < 12.53, Q4≥12.53; Calcium CV: Q1<2.28, 2.28≤Q2 <3.64, 3.64≤Q3 < 5.48, Q4≥5.48.

Adjusted by age, gender, race, body mass index; heart rate, systolic blood pressure, diastolic blood pressure, respiratory rate, Simplified Acute Physiology Score, hypertension, diabetes mellitus, chronic pulmonary disease, congestive heart failure, atrial fibrillation, ventricular arrhythmia, stroke, malignant tumor, chronic kidney disease, liver disease, vasopressin, mechanical ventilation, diuretics, sodium supplementation, potassium supplementation, calcium supplementation.

Abbreviations: ICU, intensive care unit; CV, coefficient of variation; HR, hazard ratio; CI, confidence interval.

Supplementary Table S8. Sensitivity analysis of electrolytes variability selecting CV calculated within 48 hours of ICU admission

|  | **28-day ICU mortality** | | **90-day ICU mortality** | |
| --- | --- | --- | --- | --- |
|  | **HR (95% CI)** | ***P*** | **HR (95% CI)** | ***P*** |
| Continuous Sodium CV | 1.11 (1.06, 1.17) | <0.001 | 1.08 (1.03, 1.14) | 0.002 |
| Quartiles of Sodium CV |  |  |  |  |
| Q1 | *reference* |  | *reference* |  |
| Q2 | 0.97 (0.78, 1.20) | 0.769 | 0.91 (0.76, 1.10) | 0.351 |
| Q3 | 1.15 (0.93, 1.43) | 0.185 | 1.05 (0.87, 1.26) | 0.614 |
| Q4 | 1.36 (1.10, 1.68) | 0.004 | 1.18 (1.02, 1.40) | 0.041 |
| Continuous Potassium CV | 1.01 (1.00, 1.02) | 0.031 | 1.01 (1.00, 1.02) | 0.023 |
| Quartiles of Potassium CV |  |  |  |  |
| Q1 | *reference* |  | *reference* |  |
| Q2 | 0.95 (0.76, 1.17) | 0.610 | 0.97 (0.81, 1.18) | 0.820 |
| Q3 | 0.95 (0.76, 1.17) | 0.638 | 1.03 (0.85, 1.24) | 0.757 |
| Q4 | 1.12 (1.04, 1.23) | 0.013 | 1.17 (1.02, 1.38) | 0.045 |
| Continuous Calcium CV | 1.07 (1.05, 1.08) | <0.001 | 1.06 (1.05, 1.07) | <0.001 |
| Quartiles of Calcium CV |  |  |  |  |
| Q1 | *reference* |  | *reference* |  |
| Q2 | 1.45 (1.14, 1.87) | 0.003 | 1.07 (0.93, 1.22) | 0.341 |
| Q3 | 1.59 (1.25, 2.04) | <0.001 | 1.42 (1.15, 1.76) | 0.001 |
| Q4 | 2.32 (1.83, 2.94) | <0.001 | 2.22 (1.95, 2.59) | <0.001 |

Sodium CV: Q1<1.02, 1.02≤Q2 < 1.51, 1.51≤Q3< 2.11, Q4≥2.11; Potassium CV: Q1<6.01,6.01≤Q2 < 8.92, 8.92≤Q3 < 12.53, Q4≥12.53; Calcium CV: Q1<2.28, 2.28≤Q2 <3.64, 3.64≤Q3 < 5.48, Q4≥5.48.

Adjusted by age, gender, race, body mass index; heart rate, systolic blood pressure, diastolic blood pressure, respiratory rate, Simplified Acute Physiology Score, hypertension, diabetes mellitus, chronic pulmonary disease, congestive heart failure, atrial fibrillation, ventricular arrhythmia, stroke, malignant tumor, chronic kidney disease, liver disease, vasopressin, mechanical ventilation, diuretics, sodium supplementation, potassium supplementation, calcium supplementation.

Abbreviations: ICU, intensive care unit; CV, coefficient of variation; HR, hazard ratio; CI, confidence interval.

Supplementary Table S9. Best hyperparameters of each machine learning model

| Classifiers | Hyperparameters | |
| --- | --- | --- |
| Light gradient boosting machine | n_estimators | 200 |
|  | max_depth | 4 |
|  | learning_rate | 0.01 |
|  | boosting_type | gbdt |
|  | objective | binary |
|  | num_leaves | 3 |
|  | colsample_bytree | 0.9424542876051492 |
|  | min_child_samples | 2 |
|  | subsample | 0.8586542609576361 |
|  | class_weight | ‘balanced’ |
|  |  |  |
| Random Forest | n_estimators | 400 |
|  | max_depth | 6 |
|  | criterion | ‘entropy’ |
|  | min_samples_leaf | 30 |
|  | random_state | 54 |
|  | class_weight | ‘balanced’ |
|  |  |  |
| Logistic Regression | C | 10 |
|  | penalty | l2 |
|  | solver | ‘saga’ |
|  | max_iter | 100 |
|  | class_weight | ‘balanced’ |
|  |  |  |
| Support Vector Machine | C | 1 |
|  | kernel | ‘poly’ |
|  | probability | True |
|  | tol | 0.0001 |
|  | gamma | ‘auto’ |
|  | class_weight | ‘balanced’ |
|  |  |  |
| Multilayer Perceptron | solver | ‘adam’ |
|  | activation | ‘relu’ |
|  | hidden_layer_sizes | (5, 5) |
|  | alpha | 0.001 |
|  | max_iter | 300 |
|  | random_state | 3 |
|  |  |  |
| K-nearest Neighbors | n_neighbors | 24 |
|  | p | 1 |
|  | metric | minkowski |
|  | weights | distance |

Supplementary Table S10. Sensitivity analysis of electrolytes variability excluding electrolytes supplementation as a covariate

|  | **28-day ICU mortality** | | **90-day ICU mortality** | |
| --- | --- | --- | --- | --- |
|  | **HR (95% CI)** | ***P*** | **HR (95% CI)** | ***P*** |
| Continuous Sodium CV | 1.11 (1.06, 1.17) | <0.001 | 1.09 (1.04, 1.14) | <0.001 |
| Quartiles of Sodium CV |  |  |  |  |
| Q1 | *reference* |  | *reference* |  |
| Q2 | 1.01 (0.80, 1.28) | 0.910 | 0.99 (0.82, 1.21) | 0.984 |
| Q3 | 1.11 (1.08, 1.39) | 0.036 | 0.98 (0.80, 1.19) | 0.842 |
| Q4 | 1.39 (1.11, 1.72) | 0.003 | 1.24 (1.04, 1.50) | 0.002 |
| Continuous Potassium CV | 1.02 (1.01, 1.03) | <0.001 | 1.02 (1.01, 1.03) | <0.001 |
| Quartiles of Potassium CV |  |  |  |  |
| Q1 | *reference* |  | *reference* |  |
| Q2 | 0.86 (0.67, 1.08) | 0.202 | 0.96 (0.78, 1.18) | 0.713 |
| Q3 | 0.98 (0.79, 1.23) | 0.920 | 1.12 (0.92, 1.37) | 0.247 |
| Q4 | 1.23 (1.02, 1.53) | 0.047 | 1.29 (1.07, 1.56) | 0.009 |
| Continuous Calcium CV | 1.07 (1.05, 1.08) | <0.001 | 1.06 (1.04, 1.07) | <0.001 |
| Quartiles of Calcium CV |  |  |  |  |
| Q1 | *reference* |  | *reference* |  |
| Q2 | 1.43 (1.11,1.84) | <0.001 | 1.15 (0.94, 1.42) | 0.169 |
| Q3 | 1.56 (1.22, 1.99) | <0.001 | 1.32 (1.08, 1.61) | 0.006 |
| Q4 | 2.24 (1.77, 2.84) | <0.001 | 1.72 (1.42, 2.09) | <0.001 |

Sodium CV: Q1<1.02, 1.02≤Q2 < 1.51, 1.51≤Q3 < 2.11, Q4≥2.11; Potassium CV: Q1<6.01,6.01≤Q2 < 8.92, 8.92≤Q3 < 12.53, Q4≥12.53; Calcium CV: Q1<2.28, 2.28≤Q2 <3.64, 3.64≤Q3 < 5.48, Q4≥5.48.

Adjusted by age, gender, race, body mass index; heart rate, systolic blood pressure, diastolic blood pressure, respiratory rate, Simplified Acute Physiology Score, hypertension, diabetes mellitus, chronic pulmonary disease, congestive heart failure, atrial fibrillation, ventricular arrhythmia, stroke, malignant tumor, chronic kidney disease, liver disease, vasopressin, mechanical ventilation, diuretics.

Abbreviations: ICU, intensive care unit; CV, coefficient of variation; HR, hazard ratio; CI, confidence interval. interval.
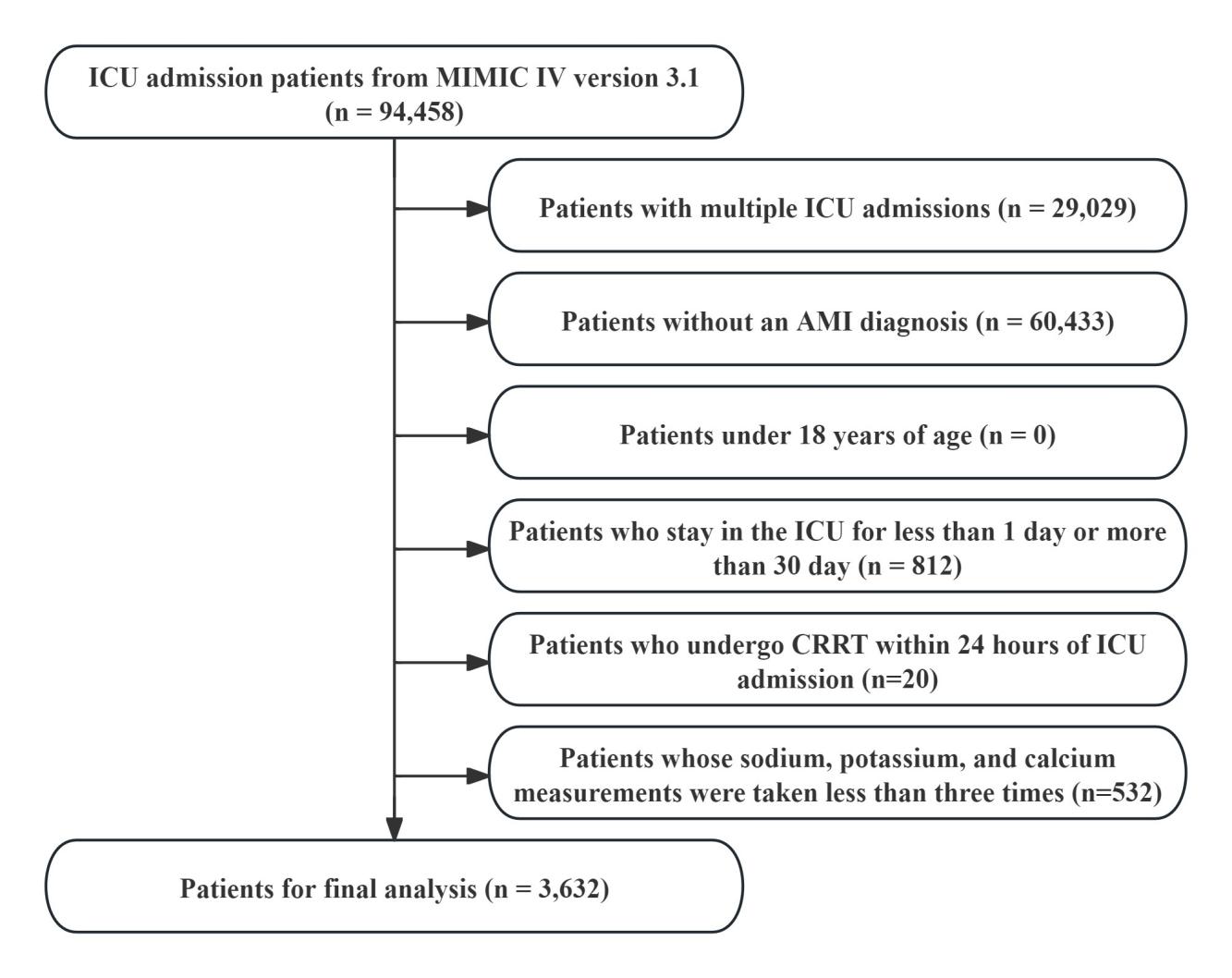


Supplementary Figure S1. Flowchart of the study population. MIMIC-IV, Medical Information Mart for Intensive Care; ICU, intensive care unit; AMI, acute myocardial infarction; CRRT, continuous renal replacement therapy.


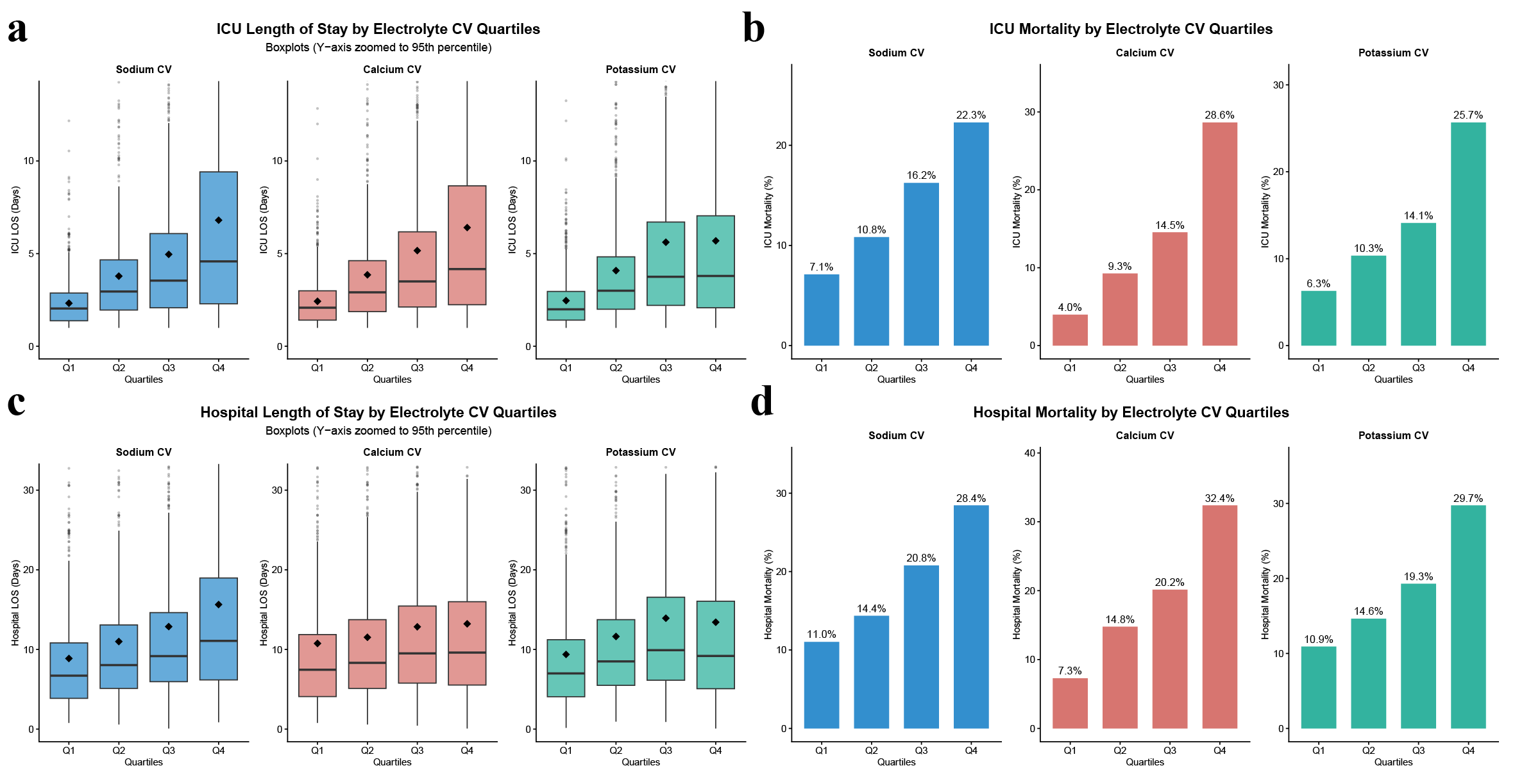


Supplementary Figure S2. ICU and hospital length of stay and mortality across electrolyte CV quartiles (Sodium, Calcium, Potassium). ICU, intensive care unit; CV, Coefficient of variation.


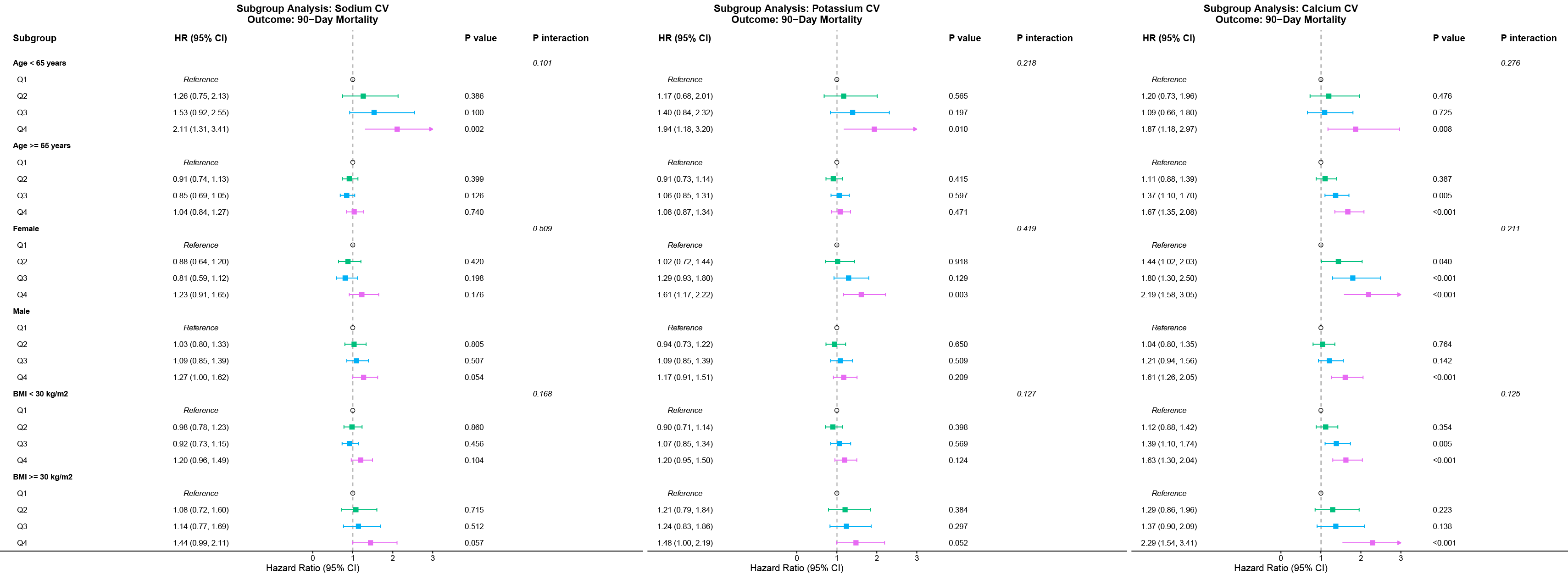


Supplementary Figure S3. Subgroup forest plots of 90-day mortality by electrolyte CV quartiles (Sodium, Potassium, Calcium). CV, Coefficient of variation; HR, hazard ratio; CI, confidence interval.

Supplementary Figure S4. Boruta feature importance ranking for predicting 28-day ICU mortality in patients with acute myocardial infarction. AF, atrial fibrillation; BMI, body mass index; CKD, chronic kidney disease; CPD, chronic pulmonary disease; DBP, diastolic blood pressure; DM, diabetes mellitus; HF, heart failure; HR, heart rate; LD, liver disease; MT, malignant tumour; MV, mechanical ventilation; RR, respiratory rate; SBP, systolic blood pressure; VA, ventricular arrhythmia.


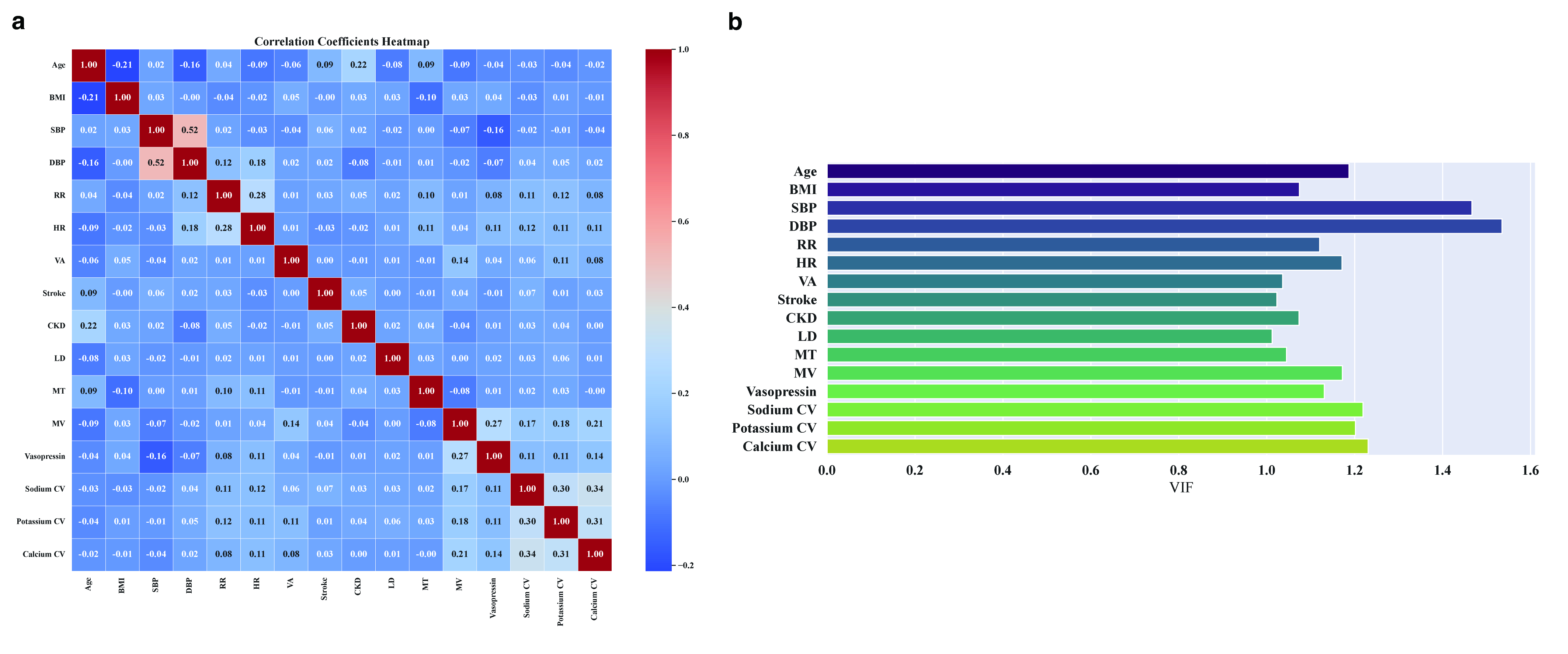


Supplementary Figure S5. Pearson’s correlation test (a) and variance inflation factor test (b) for selected features. BMI, body mass index; CKD, chronic kidney disease; DBP, diastolic blood pressure; HR, heart rate; LD, liver disease; MT, malignant tumour; MV, mechanical ventilation; RR, respiratory rate; SBP, systolic blood pressure; VA, ventricular arrhythmia; VIF, variance inflation factor.
